# Quality of life assessment in individuals with schizophrenia: a COSMIN-based systematic review of S-QoL41 and S-QoL18 incorporating neurobiological support

**DOI:** 10.1016/j.eclinm.2026.104152

**Published:** 2026-08-15

**Authors:** Masoud Rahmati, Marianne Foiselle, Sara Fernandes, Bach Xuan Tran, Lee Smith, Dong Keon Yon, Jean-Philippe Suppini, Jonathan Chelly, Manuel Dias Alves, Pascal Auquier, Karine Baumstarck, Laurent Boyer

**Affiliations:** a CEReSS-Health Service Research and Quality of Life Center, Aix-Marseille University, Marseille, France; b Clinical Research Unit, Delegation a la Recherche Clinique et a l’Innovation du GHT, Centre Hospitalier Intercommunal Toulon La Seyne sur-Mer, Toulon, France; c CEReSS-Health Service Research and Quality of Life Center, Assistance Publique-Hopitaux de Marseille, Aix-Marseille University, Marseille, France; d Faculty of Public Health, VNU University of Medicine and Pharmacy, Vietnam National University, Hanoi, Vietnam; e International Institute for Training and Research (INSTAR), VNU University of Medicine and Pharmacy, Vietnam National University, Hanoi, Vietnam; f Centre for Health, Performance, and Wellbeing, Anglia Ruskin University, Cambridge, UK; g Department of Public Health, Faculty of Medicine, Biruni University, Istanbul, Turkey; h Center for Digital Health, Medical Science Research Institute, Kyung Hee University Medical Center, Kyung Hee University College of Medicine, Seoul, Republic of Korea; i Department of Pediatrics, Kyung Hee University College of Medicine, Seoul, Republic of Korea

**Keywords:** Schizophrenia, Quality of life, Patient-reported outcome measures, COSMIN systematic review

## Abstract

**Background:**

Quality of life is a key outcome in schizophrenia, requiring valid patient-reported outcome measures. We evaluated Schizophrenia Quality of Life (S-QoL) instruments.

**Methods:**

We searched PubMed, PsycINFO, and Web of Science from inception to Dec 10, 2025. Measurement properties were assessed using the COnsensus-based Standards for the selection of health Measurement INstruments (COSMIN) guidelines and Risk of Bias checklist, and evidence quality was graded using the Grading of Recommendations Assessment, Development and Evaluation (GRADE) approach. Heterogeneity was addressed through COSMIN-based best-evidence synthesis. Neuroimaging studies were reviewed to contextualise the neurobiological basis of S-QoL domains. This review is registered with PROSPERO, CRD420261287006.

**Findings:**

Fourteen studies demonstrated sufficient validity (content, structural, and construct), internal consistency, and responsiveness of S-QoL instruments. Reliability was supported for S-QoL41 and S-QoL18, but limited for S-QoL-MCAT. Evidence for cross-cultural validity and measurement invariance ranged from very low to moderate certainty, while evidence for measurement error was scarce. Neuroimaging findings supported the construct coherence of S-QoL domains.

**Interpretation:**

S-QoL41, S-QoL18, and S-QoL-MCAT demonstrate robust psychometric properties and are suitable for clinical research and practice. S-QoL18 offers the best balance between psychometric performance and feasibility. Further research should address cross-cultural validity, measurement error, and thresholds for individual-level change.

**Funding:**

There was no specific funding source for this research.

**Research in context**

**Evidence before this study**

Health-related quality of life (QoL) is a core patient-reported outcome in schizophrenia, reflecting dimensions of well-being and functioning that extend beyond symptom severity. The Schizophrenia Quality of Life instruments (S-QoL41, S-QoL18, and S-QoL-MCAT) are among the most widely used schizophrenia-specific quality-of-life measures and have been applied across diverse populations and settings. We searched PubMed, PsycINFO, and Web of Science from database inception to 10 December 2025 using terms related to schizophrenia, quality of life, S-QoL instruments, and measurement properties, including studies published in English. The search identified 14 eligible studies. Evidence regarding the measurement properties of S-QoL instruments has remained fragmented across individual validation studies, and the available evidence has not previously been synthesised using the COnsensus-based Standards for the selection of health Measurement INstruments (COSMIN) framework.

**Added value of this study**

We conducted the first COSMIN-based systematic review of the S-QoL41, S-QoL18, and S-QoL-MCAT, including 14 studies and 2555 participants from six countries. We systematically evaluated content validity, structural validity, internal consistency, reliability, construct validity, responsiveness, cross-cultural validity, and measurement error, and graded the certainty of evidence using the GRADE approach. The review demonstrated predominantly sufficient evidence for content validity, structural validity, internal consistency, construct validity, and responsiveness, with S-QoL18 showing the most favourable balance between psychometric performance and feasibility. In addition, we synthesised neurobiological evidence linking S-QoL scores to brain structure and function, providing complementary contextual support for the theoretical coherence and clinical relevance of the constructs captured by these instruments.

**Implications of all the available evidence**

The available evidence supports the use of S-QoL instruments, particularly S-QoL18, as robust patient-reported measures of quality of life in schizophrenia research and clinical practice.

Beyond demonstrating favourable psychometric performance, the convergence between patient-reported quality of life and neurobiological correlates supports that S-QoL instruments capture clinically relevant dimensions of living with schizophrenia. Future research should strengthen evidence on cross-cultural validity, measurement error, and meaningful within-person change, while further investigating the neurobiological mechanisms underlying QoL outcomes in schizophrenia.

## Introduction

Schizophrenia is a severe and disabling mental disorder that imposes extensive physical, psychological, and social burdens, profoundly affecting quality of life (QoL).^1^, ^2^, ^3^ Subjective QoL may also be influenced by emotional processing deficits, alexithymia, and patients’ perceived treatment experiences, which are important considerations when interpreting patient-reported outcomes in schizophrenia.^4^ Measuring QoL provides insights beyond clinical symptoms, helping clinicians and researchers identify intervention targets, evaluate treatment effects, and understand determinants of well-being.^5^

The Schizophrenia Quality of Life questionnaire (S-QoL41) is a schizophrenia-specific patient-reported outcome measure (PROM) developed from patients’ perspectives to assess quality of life and recovery-related outcomes in schizophrenia.^1^ A shortened version (S-QoL18) was subsequently developed to reduce respondent burden,^2^ and a computerized adaptive version (S-QoL-MCAT) was introduced to improve assessment efficiency while maintaining precision.^6^ These instruments are used to support treatment evaluation, outcome monitoring, and patient-centred care.^1^^,^^2^

Despite their widespread use in people with schizophrenia, including those with severe cognitive impairments, homeless individuals, and inpatients, and their availability in multiple languages,^1^^,^^2^^,^^6^, ^7^, ^8^, ^9^, ^10^, ^11^, ^12^, ^13^, ^14^, ^15^, ^16^ no systematic review has comprehensively evaluated the psychometric properties of the S-QoL41, S-QoL18, and S-QoL-MCAT instruments. Although derived from the same conceptual framework, differences in instrument length and administration format may affect their psychometric performance and clinical utility, necessitating a comprehensive evaluation of their measurement properties to inform instrument selection. Using the Consensus-based Standards for the Selection of Health Measurement Instruments (COSMIN) methodology, an internationally recognised framework for evaluating PROMs alongside its Risk of Bias Checklist, we provide a standardised assessment of these instruments. To our knowledge, this is the first comprehensive evaluation of all three S-QoL instruments. Neurobiological findings are additionally discussed as complementary contextual evidence to aid interpretation of the clinical and theoretical relevance of the constructs assessed by these instruments. Outside the scope of COSMIN appraisal, these findings are considered exploratory and hypothesis-generating rather than evidence of construct validity, with potential relevance for future mechanistic research. By synthesising evidence on validity, reliability, responsiveness, and interpretability, this review identifies the strengths and limitations of each instrument and informs the selection of robust, person-centred measures of QoL for both clinical practice and research.

## Methods

This systematic review was carried out following the COSMIN guideline for systematic reviews of PROMs (version 2.0, 2024 update), and reported following the PRISMA-COSMIN for Outcome Measurement Instruments reporting guideline.^17^^,^^18^ The review protocol was prospectively registered in the International Prospective Register of Systematic Reviews (PROSPERO; CRD420261287006). A comprehensive summary of the methodological framework is provided in Table 1.

**Table 1:** Application of the COSMIN methodology to S-QoL.

Evaluation stage	Analytical component	Purpose of assessment	Outcome classification
Stage 1. Evidence identification	Systematic literature screening	Identification of all studies reporting measurement properties of S-QoL instruments	Eligible studies selected
Stage 2. Methodological appraisal	COSMIN Risk of Bias tool	Appraisal of methodological rigour using a four-level rating system	Very good/Adequate/Doubtful/Inadequate
	Instrument development	Assessment of construct definition, conceptual framework, target population, context of use, and qualitative development procedures	Qualitative judgement
	Content validity	Evaluation of relevance, comprehensiveness, and comprehensibility from patient and expert perspectives	Qualitative judgement
	Structural validity	Examination of factor structure or IRT/Rasch model fit and sample adequacy	Qualitative judgement
	Internal consistency	Assessment of inter-item coherence (Cronbach's alpha), conditional on sufficient structural validity	Qualitative judgement
	Cross-cultural validity/Measurement invariance	Evaluation of translation procedures and measurement invariance across language or cultural groups	Qualitative judgement
	Reliability	Stability of scores over time or between raters using ICC estimates	Qualitative judgement
	Measurement error	Analysis of measurement error indices relative to meaningful change thresholds	Qualitative judgement
	Criterion validity	Correlation with an established reference standard, when available	Qualitative judgement
	Hypotheses testing for construct validity	Testing predefined hypotheses for convergent and known-groups validity	Qualitative judgement
	Responsiveness	Ability to detect change over time using hypothesis-based or subgroup approaches	Qualitative judgement
Stage 3. Evaluation of measurement performance	COSMIN criteria for good measurement properties	Judgement of each measurement property	Sufficient (+)/Insufficient (−)/Inconclusive (?)
Stage 4. Certainty of evidence	Modified GRADE approach	Overall confidence in the evidence considering bias, inconsistency, and imprecision	High/Moderate/Low/Very low
Stage 5. Instrument recommendation	PROM endorsement decision	Classification of S-QoL instruments for use in research or practice	PROM recommendation based on evidence synthesis

COSMIN, Consensus-based Standards for the selection of health Measurement INstruments; S-QoL, Schizophrenia Quality of Life; PROM, Patient-Reported Outcome Measure; IRT, Item Response Theory; ICC, Intraclass Correlation Coefficient; GRADE, Grading of Recommendations Assessment, Development and Evaluation.

### Search strategy and selection criteria

A comprehensive literature search was conducted across PubMed, PsycINFO, and Web of Science from database inception through 10 December 2025. The search strategy was developed in accordance with COSMIN recommendations for systematic reviews of PROMs and was structured around the target construct, instrument, and measurement properties. The search strategy combined terms for the schizophrenia-related QoL, S-QoL instruments (including S-QoL18, S-QoL41, S-QoL-MCAT, and related variants) with keywords related to measurement properties. The detailed search queries for each database are provided in Supplementary Table S1. Two reviewers (MR and MF) independently screened all records, and discrepancies were resolved by discussion with additional reviewers (LB and PA). Additionally, reference lists of included studies were manually checked to identify further relevant publications.^19^ No language restrictions were applied as long as an English abstract was available.

Eligibility criteria were established based on COSMIN guidelines for systematic reviews of PROMs. We included empirical studies that evaluated at least one measurement property of the S-QoL18, S-QoL41 or S-QoL-MCAT in populations with schizophrenia. There were no restrictions regarding participants’ age, clinical setting, or language of administration. All validated formats of the instruments were considered eligible, whether self-reported, interviewer-administered, or proxy-reported. Although neuroimaging studies are not part of the COSMIN taxonomy for measurement properties, studies conducted by the original instrument developers that employed neuroimaging or related imaging modalities were additionally identified and reviewed. These studies were considered as providing neurobiological support for the theoretical constructs underlying the S-QoL dimensions, rather than as evidence of formal psychometric properties. Their inclusion aimed to contextualise the conceptual robustness of the instruments and to illustrate potential neurobiological coherence between subjective QoL domains and objective brain correlates, without contributing to the COSMIN-based evaluation or evidence synthesis.

Non-original publications (e.g., narrative reviews, editorials, conference abstracts) and studies evaluating only other QoL instruments without S-QoL18 or S-QoL41 were excluded; however, reference lists of narrative reviews were screened to identify additional relevant studies.

Screening of titles, abstracts, and full texts was conducted independently by two reviewers (MR and MF). Discrepancies were resolved through discussion, with additional reviewers (LB and PA) consulted if consensus could not be reached.

Data were independently extracted by two reviewers (MR and MF) using standardized COSMIN forms. Extracted information included study characteristics such as author, year, country, population characteristics, sample size, mean age (SD), and language of administration.

In addition, details on measurement properties were collected, content validity, including structural validity (factor analysis), internal consistency, cross-cultural validity, reliability (intraclass correlation, Pearson, or Spearman coefficients), measurement error (standard error of measurement, smallest detectable change), construct validity (e.g., convergent validity), responsiveness, and floor and ceiling effects. Information required for evidence synthesis and grading was also collected where available. Discrepancies between reviewers were resolved by consensus, or, when necessary, in consultation with additional reviewers (LB and PA).

### Evaluation of the quality of studies and PROMs

The evaluation of measurement properties was conducted in accordance with the COSMIN framework, encompassing the Risk of Bias (RoB) checklist, the updated criteria for good measurement properties, grading the quality of evidence, and development of recommendations. Moreover, the lack of a recognised gold standard precluded assessment of criterion validity. These assessments were performed independently by two reviewers (MR and MF), and any disagreements were resolved through discussion with additional reviewers (LB and PA).

The measurement properties of all PROMs were assessed individually in accordance with the recommended sequence outlined in the COSMIN guidelines. Initially, content validity was evaluated for each PROM. In addition to studies specifically reporting on content validity, we also retrieved and considered information related to the development process of PROMs.

Each study was first assessed using the COSMIN Risk of Bias checklist, with methodological quality rated as very good, adequate, doubtful, or inadequate. In line with COSMIN guidance, only critical methodological flaws were considered sufficient to downgrade risk of bias, thereby avoiding over-penalisation of minor limitations. The distinction between standards and criteria was carefully applied when interpreting results. Subsequently, each PROM was evaluated according to COSMIN criteria for good measurement properties, resulting in ratings of sufficient (+), insufficient (−), or indeterminate (?) (Supplementary Table S2).

The results from all available studies for each measurement property were then synthesized at the PROM level and judged against the same criteria. When findings across studies were consistent, they were summarized as sufficient (+), insufficient (−), or indeterminate (?). In cases of inconsistency between study results, COSMIN recommendations for handling heterogeneity were followed, which led either to an inconsistent (±) rating or to separate ratings for relevant subgroups of studies (+, −, ?). Indeterminate findings were excluded from the summary when other studies reported sufficient or insufficient results. The final stage of evaluation for each measurement property and PROM involved grading the overall quality of evidence using the GRADE approach. Evidence quality was categorized as high, moderate, low, or very low, based on considerations of risk of bias, inconsistency, imprecision, and indirectness.^17^^,^^20^ To assess construct validity and responsiveness, predefined hypotheses were established a priori and subsequently compared with the empirical findings for each PROM, as presented in Supplementary Tables S3 and S4, respectively.^1^^,^^2^^,^^6^, ^7^, ^8^, ^9^, ^10^, ^11^, ^12^, ^13^, ^14^, ^15^, ^16^^,^^21^^,^^22^

### Data synthesis and analysis

Owing to the availability of several language versions and the substantial heterogeneity in study populations, instrument versions, outcome definitions, and statistical indices reported across studies, conducting a pooled meta-analysis of internal consistency (Cronbach's alpha) and test–retest reliability (ICC) was not methodologically justified. Alternative quantitative synthesis approaches were considered; however, the degree of clinical, methodological, and statistical heterogeneity across studies was judged to be too substantial to support meaningful aggregation of estimates. Instead, a structured best-evidence synthesis was undertaken, incorporating risk of bias, methodological quality, and consistency of findings. Results were summarised narratively and stratified by measurement property (reliability, validity, and responsiveness) and instrument type. In accordance with COSMIN recommendations. Instead, results were descriptively reported by presenting the range of values for each instrument, providing an overall summary of the available evidence.^17^

### Formulation of recommendations

Based on the overall evaluation of content validity, other measurement properties, and the quality of evidence, a final conclusion was drawn regarding the suitability of each PROM for use. PROMs supported by sufficient content validity and adequate evidence for relevant measurement properties were considered appropriate for recommendation, whereas instruments with high-quality evidence of insufficient measurement properties were not recommended. When the available evidence did not allow a clear judgement, no definitive recommendation was made. Recommendations for future research were formulated with a focus on the most promising PROMs. If more than one instrument was considered suitable for use, feasibility and interpretability were additionally taken into account to identify the most appropriate PROM.^17^

### Ethics statement

Current study was performed based on previously published studies. The ethical approval and informed consent were not required.

### Role of the funding source

No funding was received for this work.

## Results

### Study characteristics

**Fig. 1:**
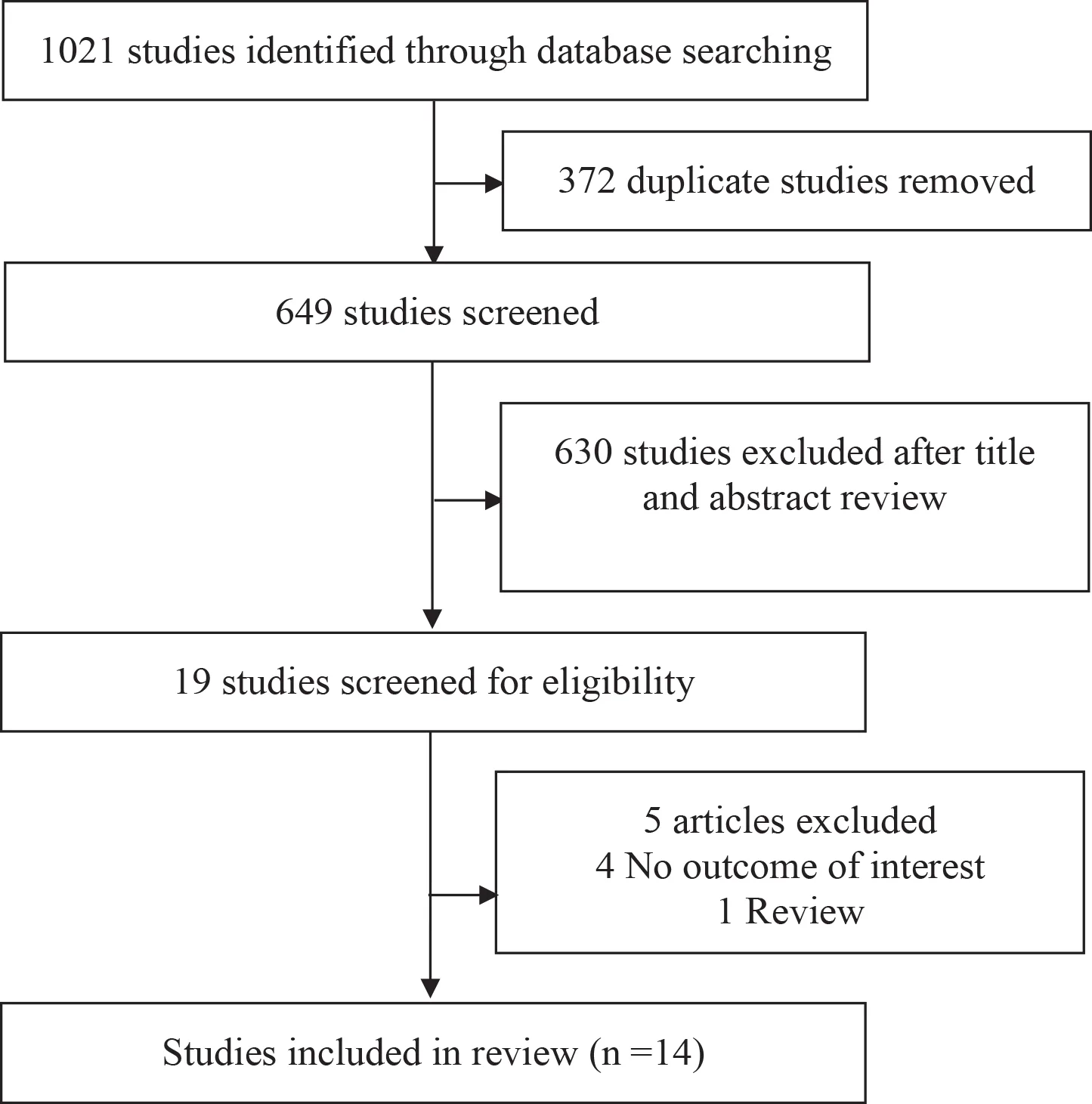
Preferred Reporting Items for Systematic Reviews and Meta-Analyses (PRISMA) flow diagram of study selection.

A total of 1021 articles were initially identified through the literature search. Following removal of duplicates and screening of titles and abstracts, 14 studies were retained for full-text review (see Supplementary Table S5 and Fig. 1). These studies, encompassing approximately 2555 participants, assessed the measurement properties of the S-QoL18, S-QoL41, and S-QoL-MCAT across diverse populations with schizophrenia and across multiple geographic regions.

A summary of the general characteristics of the included studies is presented in Table 2. The 14 included studies encompassed a range of populations with schizophrenia, including homeless individuals and patients with cognitive impairment, across multiple countries (France, Taiwan, Morocco, Bolivia, Chile, and Peru). Sample sizes ranged from 41 to 517 participants, ages varied from 29.8 to 38.6 years on average, and the instruments were primarily self-administered, with some studies using mixed administration modes. Languages of administration included French, Spanish, Chinese, and Moroccan Arabic. Across studies, samples were predominantly male (70%), with reported proportions of male participants ranging from 58% to 100%.

**Table 2:** General characteristics of included individuals.

Author	Country	Population	Sample (%male/%female)	Age (years), mean (SD)	Language	PROM
Auquier, 2003^1^	France	Schizophrenia	207 (70/30)	37.3 (10.9)	French	S-QoL41
Reine, 2005^16^	France	Schizophrenia	207 (68/32)	37.4 (10.9)	French	S-QoL41
Boyer, 2010^2^	France	Schizophrenia	517 (70/30)	36.5 (10.8)	French	S-QoL18
Chou, 20111^2^	Taïwan	Schizophrenia	41 (70/30)	37.9 (7.36)	Chinese	S-QoL41
Auquier, 2013^7^	France	Homeless individuals with Schizophrenia	236 (58/42)	37.9 (10.8)	French	S-QoL18
Baumstark, 2013^8^	France	Individuals with Schizophrenia and cognitive impairment	113 (70/30)	38.6 (10.8)	French	S-QoL18
Baumstark, 2014^9^	France	Individuals with Schizophrenia and cognitive impairment	113 (70/30)	38.6 (10.8)	French	S-QoL18
Caqueo-Urizar, 2014^11^	Bolivia, Chile, Peru	Schizophrenia	253 (65/35)	Bolivia: 33.7 (11.3) Chile: 37.9 (15.3) Peru: 35.2 (10.3)	Spanish	S-QoL18
Chou, 2015^13^	Taïwan	Schizophrenia	41 (60/40)	37.9 (7.36)	Chinese	S-QoL18
Faget-Agius, 2015^23^	France	Schizophrenia	81 (100/0)	30 (7.5)	French	S-QoL18
Faget-Agius, 2016^24^	France	Schizophrenia	131 (73/27)	35.8 (11)	French	S-QoL18
Girard, 2016^25^	France	Homeless individuals with Schizophrenia	55 (72/28)	<30	French	S-QoL41
Michel, 2018^6^	France	Schizophrenia	517 (70/30)	36.5 (10.9)	French	MCAT
Kachouchi, 2026^26^	Morocco	Schizophrenia	43 (74/26)	29.8 (8.07)	Moroccan Arabic	S-QoL18

PROM, Patient-Reported Outcome Measure; S-QoL, Schizophrenia Quality of Life instruments; MCAT, multidimensional computerized adaptive test; SD, Standard Deviation.

### PROM development and content validity of Schizophrenia Quality of Life questionnaire (S-QoL)

The Schizophrenia Quality of Life questionnaire (S-QoL41) was developed using a patient-centred approach based on Calman's HRQoL model, which defines QoL as the gap between expectations and lived experience.^1^ Initial items were generated through interviews with 20 patients of varying clinical profiles, leading to 97 items after achieving conceptual saturation. Pretesting in 40 patients reduced the pool to 85 items. Further refinement using expert review and psychometric analyses, including Rasch modelling, ensured unidimensionality. The final instrument includes 41 items across eight dimensions: psychological well-being, self-esteem, relationships, resilience, physical well-being, autonomy, and sentimental life. Cognitive interviews further optimised clarity, relevance, and response formats prior to large-scale validation.^1^

The shortened version (S-QoL18) was developed using pooled data from four multicentre studies (n = 517). Item reduction combined classical test theory, item response theory, and expert review to maintain conceptual coverage while reducing burden. Differential item functioning (DIF) analyses confirmed stability across demographic and clinical subgroups.^2^ Additional studies in patients with cognitive impairments supported preserved dimensionality, item relevance, and overall comprehensibility.^8^^,^^9^

The computerized adaptive version (S-QoL-MCAT) was derived entirely from the S-QoL41 item bank, with no new items or domains. Item wording was unchanged; only item selection and order were adapted, with a mean of 25 items administered (SD = 5; SEM < 0.55). The original eight-factor structure was confirmed using multidimensional item response theory, supporting preserved content validity consistent with the paper-based versions^6^ (Supplementary Table S6). Findings on each measurement properties are also summarised in Supplementary Table S7.

### Structural validity

The original eight-factor structure of S-QoL41 was consistently supported across studies. Auquier et al., (2003)^1^ identified an eight-factor solution explaining approximately 52% of the total variance, and subsequent confirmatory factor analysis (CFA) confirmed adequate model fit (root mean square error of approximation [RMSEA] = 0.035, comparative fit index [CFI] = 0.95, goodness-of-fit index [GFI] = 0.99, and standardized root mean square residual [SRMR] = 0.015). Similar results were reported for the computerized adaptive version, with retention of the original dimensions and acceptable fit indices (RMSEA = 0.05, CFI = 0.95).^6^

For S-QoL18, structural validity was supported by factor analyses, Rasch modelling, and item-fit analyses. Rasch INFIT statistics were within acceptable ranges across all eight dimensions, supporting unidimensionality and adequate item functioning.^2^^,^^7^, ^8^, ^9^^,^^11^ The eight-factor structure was also maintained in patients with executive dysfunction and broader cognitive impairment, with acceptable CFA and item-fit results.^8^^,^^9^ For S-QoL-MCAT, multidimensional item response theory and CFA likewise supported the original eight-factor structure (RMSEA = 0.05, CFI = 0.95).^6^

### Internal consistency

Internal consistency was evaluated across multiple studies and populations. For S-QoL41, Cronbach's α ranged from 0.72 to 0.95 for total scores and from 0.54 to 0.93 for subscales, with the lowest values reported for Self-esteem (α = 0.54) and Autonomy (α = 0.64) in smaller samples.^1^^,^^12^^,^^14^^,^^16^ For S-QoL18, total score Cronbach's α ranged from 0.72 to 0.90, and most subscales showed α ≥ 0.70, although lower values were reported for Resilience (α < 0.50) and Self-esteem (α = 0.51) in specific samples.^2^^,^^7^, ^8^, ^9^^,^^11^^,^^21^^,^^26^ Comparable Cronbach's α coefficients were observed among patients with executive dysfunction and broader cognitive impairment.^8^^,^^9^ For S-QoL-MCAT, empirical marginal reliability coefficients ranged from 0.80 to 0.92 across dimensions.^6^

### Cross-cultural validity/measurement invariance (COSMIN)

Evidence for cross-cultural validity was limited for S-QoL41, as most studies did not assess differential item functioning (DIF) or measurement invariance.^1^^,^^12^^,^^14^^,^^16^^,^^22^ For S-QoL18, DIF was identified across gender, age, education, or clinical form,^2^ whereas item functioning was comparable between homeless and non-homeless participants.^7^ One study identified DIF for a single item (“plan for future”) across countries,^11^ while another confirmed content validity following translation into Moroccan Arabic.^26^ The Chinese version retained the original eight-factor structure, although DIF was not assessed.^13^ For S-QoL-MCAT, negligible DIF was observed in only 6 of 123 tests, supporting invariance across gender, insight, and paranoia.^6^

### Reliability

Test–retest reliability was assessed for S-QoL41 and S-QoL18. For S-QoL41, intraclass correlation coefficients (ICCs) ranged from 0.64 to 0.76 across subscales, with an ICC of 0.79 for the total S-QoL Index.^1^ Similar results were reported by Chou (2011), with ICCs ranging from 0.64 to 0.87,^12^ and significant test–retest correlations over 7–30 days were also observed.^22^

For S-QoL18, ICCs ranged from 0.50 to 0.78 across subscales, with an ICC of 0.73 for the total Index.^2^ Chou (2015) reported ICCs ranging from 0.58 to 0.84 across subscales and 0.87 for the total Index.^13^ For S-QoL-MCAT, test–retest reliability was not assessed.^6^ Inter-rater reliability was not evaluated in any study.

### Measurement error

No study formally evaluated measurement error according to COSMIN criteria. Although Michel et al., (2018)^6^ reported RMSE and standard error of measurement (SEM) for S-QoL-MCAT, these estimates reflected score precision within the item response theory/computerized adaptive testing framework rather than measurement error as defined by COSMIN. No studies reported measurement error parameters such as SEM, smallest detectable change (SDC), minimal detectable change (MDC), or limits of agreement.

### Construct validity (hypotheses testing)

For convergent validity, correlations between S-QoL instruments and QoL measures, recovery measures, self-esteem scales, and clinical indicators were generally consistent with the predefined hypotheses for S-QoL41, S-QoL18, and S-QoL-MCAT.^1^^,^^2^^,^^6^^,^^8^^,^^9^^,^^11^, ^12^, ^13^, ^14^^,^^16^^,^^21^^,^^22^

Known-groups validity was supported by the ability of S-QoL41 and S-QoL18 to discriminate between groups differing in symptom severity, remission status, depressive symptoms, functioning, cognitive impairment, hospitalisation history, and housing status.^2^^,^^7^^,^^8^^,^^10^^,^^15^ Associations with demographic variables were generally weak or absent, as hypothesised (Supplementary Table S8). Overall, 84.5% of the predefined hypotheses across construct validity analyses were confirmed.

### Responsiveness/sensitivity to change

For S-QoL41, longitudinal studies showed changes consistent with clinical improvement, with significant improvements and small-to-moderate effect sizes (effect size [ES] ≥ 0.20) observed for the total score and key domains.^1^^,^^16^ Some domains showed smaller or non-significant changes, in line with predefined hypotheses (Supplementary Table S9). For S-QoL18, significant improvements were reported across multiple studies, particularly in Psychological Well-being, Self-esteem, Social Relationships, Sentimental Life, and total scores, with effect sizes generally meeting or exceeding the predefined threshold (ES ≥ 0.20).^2^^,^^7^^,^^13^ Group comparisons based on clinical improvement, symptom remission, and hospitalisation status were consistent with expected hypotheses. No responsiveness data were available for S-QoL-MCAT; therefore, this measurement property could not be evaluated for this version. Overall, 89% of the predefined hypotheses across responsiveness analyses were confirmed.

### Item score distribution, floor and ceiling effects

Item score distributions and floor/ceiling effects were evaluated across S-QoL instruments. For S-QoL41, floor and ceiling effects were generally low (<10–10%) across most dimensions, although higher values were reported for the Sentimental Life dimension in some studies (up to 16.9%) and with wider ranges observed across subscales in individual samples.^1^^,^^12^^,^^14^^,^^22^ For S-QoL18, floor effects were generally low (<10–20%) across most dimensions, with higher values observed in domains such as Relationships with Friends, Relationships with Family, and Sentimental Life. Ceiling effects were typically low (<10–12%), with slightly higher values in Psychological Well-being and more impaired populations.^2^^,^^7^, ^8^, ^9^^,^^11^^,^^13^ Data on S-QoL-MCAT were not available.

### Interpretability and feasibility

According to COSMIN criteria (>15–20% indicating problematic floor or ceiling effects), S-QoL41 generally showed acceptable score distributions across studies, although higher floor and ceiling effects were observed in some domains, particularly Sentimental Life and Psychological Well-being (up to 30.9% and 41.6%, respectively).^1^^,^^12^^,^^14^^,^^22^ For S-QoL18, most studies reported floor and ceiling effects below 15–20%, although higher values were observed in specific domains and populations (up to 38% and 35.1%, respectively).^2^^,^^7^, ^8^, ^9^^,^^11^^,^^13^ Floor and ceiling effects were not reported for S-QoL-MCAT. Regarding feasibility, S-QoL18 reduced respondent burden compared with S-QoL41, with shorter completion time (<5 min vs 13.6 min) and low missing data rates (2–10%).^1^^,^^2^ S-QoL-MCAT provides adaptive electronic administration with high measurement precision, although data on score distributions were not available.^6^ Information on implementation requirements (e.g., training, cost, and administrative burden) was limited across studies.

### Overall quality of evidence

Overall, S-QoL instruments demonstrated sufficient measurement properties, with strongest evidence for structural validity, internal consistency, construct validity, reliability, and responsiveness across S-QoL41, S-QoL18, and S-QoL-MCAT. Certainty of evidence was generally moderate to high for S-QoL41 and S-QoL18, and moderate for S-QoL-MCAT (Supplementary Table S10).

### Grading the quality of evidence

Most measurement properties were supported by studies of adequate methodological quality, with no downgrading for risk of bias. Evidence for cross-cultural validity and measurement error remained limited due to the absence of formal measurement invariance and measurement error analyses, and was therefore considered indeterminate. Minor inconsistencies across studies in factor structure and responsiveness led to a one-level downgrade for inconsistency. No downgrading was applied for imprecision or indirectness. Collectively, the evidence supports the use of S-QoL instruments as valid, reliable, and responsive measures of health-related quality of life in schizophrenia, while highlighting the need for further research on cross-cultural measurement properties and measurement error.

### COSMIN-based recommendation for use

According to COSMIN 2024 criteria, S-QoL41, S-QoL18, and S-QoL-MCAT can be recommended for use in people with schizophrenia. S-QoL18 provides the most favourable balance between measurement performance and feasibility, while retaining the original multidimensional structure with reduced respondent burden. S-QoL41 remains appropriate when a comprehensive assessment of quality of life domains is required, particularly in research settings. S-QoL-MCAT offers an efficient computerized adaptive alternative with high measurement precision, although some measurement properties are less extensively evaluated compared with the paper-based versions.

### Exploratory biological context

Neuroimaging evidence was additionally synthesised as a complementary and contextual layer to support the theoretical plausibility of S-QoL constructs. In a functional Single Photon Emission Computed Tomography (SPECT) study of 130 patients with schizophrenia, scores on S-QoL18 dimensions were associated with regional cerebral blood flow in areas implicated in emotion processing, social cognition, and decision-making, including Brodmann areas 6, 8, 9, 10, the striatum, and the praecuneus.^24^ Similarly, a magnetisation transfer imaging study demonstrated associations between S-QoL scores and microstructural integrity in the temporal lobes, insula, cerebellum, and occipital cortex.^23^ Given differences in imaging modalities (SPECT vs magnetisation transfer MRI), analytic approaches, and sample characteristics, these findings should be interpreted as exploratory and hypothesis-generating rather than confirmatory evidence of measurement validity. Overall, neuroimaging evidence provides complementary neurobiological support for the multidimensional QoL construct in schizophrenia.

## Discussion

The findings of this COSMIN-based review indicate that S-QoL41, S-QoL18, and S-QoL-MCAT constitute a coherent family of patient-reported outcome measures with psychometric performance that is sufficiently robust to justify their use in both clinical research and routine care for people with schizophrenia. For structural validity, internal consistency, test–retest reliability, construct validity, and responsiveness, multiple studies of adequate or very good methodological quality converge on positive findings^1^^,^^2^^,^^6^, ^7^, ^8^, ^9^, ^10^, ^11^, ^12^, ^13^, ^14^, ^15^, ^16^^,^^21^^,^^22^; accordingly, the overall certainty of evidence for these properties was graded as moderate. However, evidence for cross-cultural validity and measurement error remains limited, with few studies undertaking formal invariance testing or measurement error analysis, leading to low to very low certainty. Beyond the COSMIN-defined psychometric properties, studies employing neuroimaging reported associations between S-QoL scores and relevant brain regions, providing preliminary contextual evidence consistent with the biological relevance of the instrument's multidimensional structure.^23^^,^^24^

The findings confirm the stability of an eight-dimensional, patient-derived QoL construct across S-QoL41, S-QoL18, and S-QoL-MCAT, with clinically plausible associations to symptoms, functioning, and other QoL measures. Item reduction and computerized adaptive administration have preserved construct coverage and responsiveness. However, evidence for full cross-cultural invariance remains insufficient, with current support largely limited to structural consistency across language versions. These results reinforce a patient-centred conceptualisation of QoL in schizophrenia, rooted in Calman's expectancy-discrepancy model and developed through non-directive patient interviews that prioritised domains such as autonomy, resilience, and sentimental life beyond symptom-focused outcomes.^1^^,^^27^ Psychometrically, the consistency of the eight-factor structure across multiple settings suggests a broadly stable multidimensional construct,^11^^,^^12^^,^^14^^,^^21^ although formal equivalence across cultures cannot yet be confirmed. Studies in cognitively impaired and severely ill populations further support the robustness of S-QoL structure and item relevance across clinical severity strata.^8^^,^^9^

The S-QoL-MCAT demonstrates the application of item response theory and computerized adaptive testing to reduce respondent burden while preserving measurement precision, particularly relevant in cognitively impaired or severely symptomatic populations.^6^ The three instruments differ in their balance between measurement comprehensiveness, feasibility, and mode of administration. S-QoL18 appears to offer a favourable balance between measurement performance and feasibility, S-QoL41 provides a more comprehensive assessment when greater detail is required, and S-QoL-MCAT offers an efficient adaptive format with high measurement precision but more limited cumulative evidence. Together, these results support the use of S-QoL instruments as core measures in recovery-oriented care and research.

Functional and structural neuroimaging studies demonstrate that key dimensions, including psychological well-being, autonomy, and social relationships, are associated with activity and microstructural integrity in brain regions implicated in emotion, decision-making, and social cognition.^23^^,^^24^ These findings provide preliminary contextual evidence regarding the biological correlates of S-QoL domains. In addition, associations with functional outcomes further support clinical validity.^28^ Together, patient-derived item generation and exploratory neurobiological findings provide complementary contextual information within COSMIN's conceptual framework.^29^ Clustering analyses identifying reproducible QoL strata (high, moderate, low) further suggest meaningful latent structure aligned with clinical severity and functioning.^29^ Collectively, these data support S-QoL instruments as patient-centred measures of QoL in schizophrenia.

Beyond schizophrenia, the validity and reliability of S-QoL18 have been confirmed in other psychiatric populations. Boyer et al. (2022)^30^ demonstrated that S-QoL18 is a psychometrically sound tool for assessing QoL in patients with bipolar and depressive disorders, with the eight-factor structure confirmed via confirmatory factor analysis and significant correlations with symptomatology and functioning. Similarly, Girard et al. (2016)^25^ showed that S-QoL18 can be applied to homeless individuals with bipolar disorder or schizophrenia, maintaining adequate internal consistency, external validity, and sensitivity to change. These findings suggest potential cross-diagnostic applicability of S-QoL18, although further validation across heterogeneous settings is required. Equity and inclusion are notable features of the S-QoL programme, given that development and validation studies included individuals with severe illness, cognitive impairment, and homelessness.^7^, ^8^, ^9^^,^^14^ However, psychometric evidence remains geographically concentrated, with limited data from African, South Asian, and other under-represented regions, limiting global generalisability. Evidence from cognitively impaired and socially marginalised populations supports feasibility and structural robustness across vulnerable groups. This participatory approach aligns with rights-based and recovery-oriented frameworks and enhances the acceptability and relevance of the instruments to service users.^1^

Several limitations should be considered. Heterogeneity in study designs, sample sizes, and analytic approaches necessitated a primarily descriptive synthesis of psychometric indices, limiting precision and precluding formal exploration of statistical heterogeneity. Evidence on cross-cultural validity and measurement error was limited and frequently based on incomplete invariance testing, resulting in low to very low certainty ratings due to insufficient formal measurement invariance testing across cultural groups, limiting confidence in cross-cultural score equivalence. Consequently, cross-national and cross-group comparisons of S-QoL scores should be interpreted with caution, particularly when used for cross-country comparisons or clinical interpretation of absolute scores, as unresolved measurement invariance may bias inferences across populations. This limitation further limits the generalisability of the recommendations across different cultural and linguistic contexts. In addition, inter-rater reliability was not reported in any of the included studies; therefore, the evidence base is limited to test–retest reliability only. Criterion validity for schizophrenia-specific QoL was seldom evaluated, and the absence of a widely accepted gold standard for this construct constrained the interpretability of criterion-based assessments. Finally, data on real-world implementation and performance of the S-QoL-MCAT in routine care remain sparse, with evidence derived from one cross-sectional based on simulations rather than large-scale clinical deployment.

Future research should prioritise robust cross-cultural validation across diverse countries, languages, and socio-demographic groups, alongside longitudinal studies to clarify responsiveness and minimal important differences across treatment modalities and illness stages. Measurement error in real-world clinical contexts requires further investigation, and implementation research should explore integration of S-QoL18 and S-QoL-MCAT into routine care and digital platforms, assessing feasibility, acceptability, and impact on decision-making.^31^ In the absence of established minimal important differences or smallest detectable change, score changes should be interpreted cautiously at the individual level and in conjunction with clinical assessment and longitudinal trends, rather than relying on absolute change thresholds. Co-design with service users, carers, and clinicians will be essential to ensure that future refinements address evolving priorities such as stigma, social determinants of health, and digital connectivity.

This COSMIN-based systematic review shows that the S-QoL measurement system demonstrates overall acceptable evidence supporting its use as a patient-centred approach to assessing health-related quality of life in schizophrenia. Across studies, evidence for key measurement properties including structural validity, reliability, construct validity, and responsiveness was generally supportive, although methodological quality and completeness of evidence varied across domains.

Evidence was derived from diverse clinical populations, including individuals with cognitive impairment, severe illness, and homelessness, as well as from multiple language versions; however, geographic coverage remains limited, restricting global generalisability. Persistent uncertainties regarding cross-cultural validity and measurement error underscore the need for further high-quality studies, particularly in under-represented regions and populations. Strengthening the evidence base for S-QoL instruments and integrating rigorously evaluated patient-reported outcome measures into mental health systems may support a shift towards recovery-oriented care focused on autonomy, functioning, and subjective well-being.

## Contributors

MR and LB had full access to all of the data in the study, accessed and verified the underlying data, and take responsibility for the integrity of the data and the accuracy of the data analysis. Concept and design: MR, LB, KB, and PA. Acquisition, analysis, or interpretation of data: All authors. Drafting of the manuscript: MR and LB. Critical review of the manuscript for important intellectual content: All authors. Administrative, technical, or material support: MR, LB, KB, and PA. Supervision: LB and KB. All authors read and approved the final version of the manuscript.

## Data sharing statement

Underlying study data will be shared by the corresponding author (masoud.rahmati@univ-amu.fr) on reasonable request for academic and research purposes and is subject to data sharing agreements.

## Declaration of interests

The authors declare that they have no conflicts of interest. Manuel Dias Alves received honoraria from Janssen-Cilag for participating as a site investigator in a clinical study and for participating in a scientific committee. All authors attest that they meet the current ICMJE criteria for authorship.
