# Supplementary material for: Quality of life assessment in individuals with schizophrenia: a COSMIN-based systematic review of S-QoL41 and S-QoL18 incorporating neurobiological support
Source: eClinicalMedicine. 2026 Aug 15;99:104152. doi: 10.1016/j.eclinm.2026.104152 (PMC13499451; doi:10.1016/j.eclinm.2026.104152)
Supplement: Supplementary file 1 [file mmc1.pdf]

Supplementary Materials

Quality of life assessment in individuals with schizophrenia: a COSMIN-based systematic review of S-QoL41 and S-QoL18 incorporating neurobiological support

Masoud Rahmati [1,2], Marianne Foiselle [3], Sara Fernandes[3], Bach Xuan Tran [4, 5], Lee Smith[6,7], Dong Keon Yon[8,9], Jean-Philippe Suppini [2], Jonathan Chelly[1,2], Manuel Dias Alves [1,2], Pascal Auquier[3], Karine Baumstarck[3], Laurent Boyer [3]

[1] CEReSS-Health Service Research and Quality of Life Center, Aix-Marseille University, Marseille, France (masoud.rahmati@univ-amu.fr)

[2] Clinical Research Unit, Delegation a la Recherche Clinique et a l'Innovation du GHT, Centre Hospitalier Intercommunal Toulon La Seyne sur-Mer, Toulon, France (JFS: JEANPHILIPPE.SUPPINI@ch-toulon.fr; JC : jonathan.chelly@ch-toulon.fr; MDA: MANUEL.DIASALVES@ch-toulon.fr).

[3] CEReSS-Health Service Research and Quality of Life Center, Assistance Publique-Hopitaux de Marseille, Aix-Marseille University, Marseille, France. (MF: marianne.foiselle@gmail.com; SF: sfernandes.sara@gmail.com; LB: laurent.boyer@ap-hm.fr; PA: pascal.auquier@univ-amu.fr; KB: Karine.BAUMSTARCK@univ-amu.fr).

[4] Faculty of Public Health, VNU University of Medicine and Pharmacy, Vietnam National University, Hanoi, Vietnam (BXT: bachtran@vnu.edu.vn).

[5] International Institute for Training and Research (INSTAR), VNU University of Medicine and Pharmacy, Vietnam National University, Hanoi, Vietnam.

[6] Centre for Health, Performance, and Wellbeing, Anglia Ruskin University, Cambridge, UK, (Lee.Smith@aru.ac.uk).

[7] Department of Public Health, Faculty of Medicine, Biruni University, Istanbul, Turkey.

[8] Center for Digital Health, Medical Science Research Institute, Kyung Hee University Medical Center, Kyung Hee University College of Medicine, Seoul, Republic of Korea, (DKY: yonkkang@gmail.com).

[9] Department of Pediatrics, Kyung Hee University College of Medicine, Seoul, Republic of Korea.

Corresponding author:

Masoud Rahmati, Research Centre on Health Services and Quality of Life, Aix Marseille University, Marseille, France; masoud.rahmati@univ-amu.fr

Table of content

Supplementary Table 1. Literature search strategy 3

Supplementary Table 2. COSMIN Updated Criteria for Good Measurement Properties. 5

Supplementary Table 3. A priori hypotheses for construct validity of the S-QoL instruments. 7

Supplementary Table 4. A priori hypotheses for responsiveness of the S-QoL instruments. 8

Supplementary Table 5. A list of the excluded studies and reasons for their exclusion. 9

Supplementary Table 6. Characteristics of studies on PROM development and content validity..... 10

Supplementary Table 7. Results of Measurement Properties (Part A). 12

Supplementary Table 7. Results of Measurement Properties (Part B)... 23

Supplementary Table 8. Hypotheses testing for construct validity: comparisons and results.......... 27

Supplementary Table 9. Hypotheses testing for responsiveness: comparisons and results. 38

Supplementary Table 10. Summary of findings with the evidence for each measurement property for each PROM*.. 42

Supplementary Table 1. Literature search strategy

PubMed/MEDLINE (Ovid) (n=379)

("S-QoL"[tiab] OR SQoL[tiab] OR "S-QOL"[tiab] OR SQOL[tiab] OR "S-QoL 41"[tiab] OR "SQoL-41"[tiab] OR "S-QOL41"[tiab] OR SQOL41[tiab] OR "S-QoL 18"[tiab] OR "SQoL-18"[tiab] OR "S-QOL18"[tiab] OR SQOL18[tiab] OR "SQoL-MCAT"[tiab] OR "S-QoL MCAT"[tiab] OR "S-QOL MCAT"[tiab] OR "Schizophrenia Quality of Life-MCAT"[tiab] OR "Schizophrenia Quality of Life"[tiab] OR "Schizophrenia QoL"[tiab] OR "Schizophrenia-specific QoL"[tiab])

AND

("Validation Studies"[Publication Type] OR validation[tiab] OR psychometric*[tiab] OR reliab*[tiab] OR reproducib*[tiab] OR valid*[tiab] OR "measurement propert*"[tiab] OR "internal consistency"[tiab] OR Cronbach*[tiab] OR responsiveness[tiab] OR responsive*[tiab] OR "factor analys*"[tiab] OR "factor structure"[tiab] OR CFA[tiab] OR EFA[tiab] OR "confirmatory factor analysis"[tiab] OR "exploratory factor analysis"[tiab] OR "item response theory"[tiab] OR IRT[tiab] OR Rasch[tiab] OR "structural equation model*"[tiab] OR ICC[tiab] OR "intraclass correlation"[tiab] OR test-retest[tiab] OR agreement[tiab] OR "measurement error"[tiab] OR "standard error of measurement"[tiab] OR SEM[tiab] OR MDC[tiab] OR "minimal detectable change"[tiab] OR "cross-cultural"[tiab] OR "cross cultural"[tiab] OR "measurement invariance"[tiab] OR DIF[tiab] OR "differential item functioning"[tiab] OR convergent[tiab] OR discriminant[tiab] OR "known-groups"[tiab] OR "known group"[tiab] OR "hypothesis testing"[tiab] OR interpretability[tiab] OR "ceiling effect"[tiab] OR "floor effect"[tiab])

NOT

("addresses"[Publication Type] OR "biography"[Publication Type] OR "case reports"[Publication Type] OR "comment"[Publication Type] OR "directory"[Publication Type] OR "editorial"[Publication Type] OR "festschrift"[Publication Type] OR "interview"[Publication Type] OR "lectures"[Publication Type] OR "legal cases"[Publication Type] OR "legislation"[Publication Type] OR "letter"[Publication Type] OR "news"[Publication Type] OR "newspaper article"[Publication Type] OR "patient education handout"[Publication Type] OR "popular works"[Publication Type] OR "congresses"[Publication Type] OR "consensus development conference"[Publication Type] OR "consensus development conference, nih"[Publication Type] OR "practice guideline"[Publication Type])

NOT

("animals"[MeSH Terms] NOT "humans"[MeSH Terms])

PsycINFO (n=5)

1. ("S-QoL" OR SQoL OR "S-QOL" OR SQOL OR "S-QoL 41" OR "SQoL-41" OR "S-QOL41" OR SQOL41 OR "S-QoL 18" OR "SQoL-18" OR "S-QOL18" OR SQOL18 OR "SQoL-MCAT" OR "S-QoL MCAT" OR "S-QOL MCAT" OR "Schizophrenia Quality of Life-MCAT" OR "Schizophrenia Quality of Life" OR "Schizophrenia QoL" OR "Schizophrenia-specific QoL").ti,ab.
2. (validation OR psychometric* OR reliab* OR reproducib* OR valid* OR "measurement propert*" OR "internal consistency" OR Cronbach* OR responsiveness OR responsive* OR "factor analys*" OR "factor structure" OR CFA OR EFA OR "confirmatory factor analysis" OR "exploratory factor analysis" OR "item response theory" OR IRT OR Rasch OR "structural equation model*" OR ICC OR "intraclass correlation" OR test-retest OR agreement OR "measurement error" OR "standard error of measurement" OR SEM OR MDC OR "minimal detectable change" OR "cross-cultural" OR "cross cultural" OR "measurement invariance" OR DIF OR "differential item functioning" OR convergent OR discriminant OR "known-groups" OR "known group" OR "hypothesis testing" OR interpretability OR "ceiling effect" OR "floor effect").ti,ab.
3. 1 AND 2
4. NOT (editorial OR letter OR comment OR news).pt.

5. 3 NOT 4
**Web of science (n=637)**<br>TS=(("S-QoL" OR SQoL OR "S-QOL" OR SQOL OR "S-QoL 41" OR "SQoL-41" OR "S-QOL41" OR SQOL41 OR "S-QoL 18" OR "SQoL-18" OR "S-QOL18" OR SQOL18 OR "SQoL-MCAT" OR "S-QoL MCAT" OR "S-QOL MCAT" OR "Schizophrenia Quality of Life-MCAT" OR "Schizophrenia Quality of Life" OR "Schizophrenia QoL" OR "Schizophrenia-specific QoL"))<br>AND<br>TS=(validation OR psychometric* OR reliab* OR reproducib* OR valid* OR "measurement propert*" OR "internal consistency" OR Cronbach* OR responsiveness OR responsive* OR "factor analys*" OR "factor structure" OR CFA OR EFA OR "confirmatory factor analysis" OR "exploratory factor analysis" OR "item response theory" OR IRT OR Rasch OR "structural equation model*" OR ICC OR "intraclass correlation" OR test-retest OR agreement OR "measurement error" OR "standard error of measurement" OR SEM OR MDC OR "minimal detectable change" OR "cross-cultural" OR "cross cultural" OR "measurement invariance" OR DIF OR "differential item functioning" OR convergent OR discriminant OR "known-groups" OR "known group" OR "hypothesis testing" OR interpretability OR "ceiling effect" OR "floor effect")

Supplementary Table 2. COSMIN Updated Criteria for Good Measurement Properties.

**Measurement property**	**Rating**	**Criteria**
Content validity	+	Included items are relevant for the construct, target population, and context of use, and response options and recall period are appropriate<br>*AND*<br>No key concepts are missing<br>*AND*<br>PROM items and response options are appropriately worded and PROM instructions, items and response options understood by the population of interest as intended
	?	Not enough information reported
	-	Included items are not relevant for the construct or target population<br>*OR*<br>Key concepts are missing<br>*OR*<br>PROM items and response options are not appropriately worded or not understood by the population of interest as intended
Structural validity	+	**CTT:**<br>EFA/PCA: factor loadings of each item on its factor ≥0.30<br>*AND*<br>Maximum 10% of the items have factor loadings ≥0.30 on multiple factors<br>*AND*<br>Explained variance ≥50% and structure is in line with the theory about the construct to be measured *OR* results on scree plot or Kaiser criterion (Eigenvalues >1) are in line with the theory about the construct to be measured<br>CFA: CFI or TLI or comparable measure >0.95 *OR* RMSEA <0.06 *OR* SRMR <0.08<br>**IRT/Rasch:**<br>No violation of unidimensionality: CFI or TLI or comparable measure >0.95 *OR* RMSEA <0.06 *OR* SRMR <0.08<br>*AND*<br>No violation of local independence: residual correlations among the items after controlling for dominant factor <0.20 *OR* Q3s <0.37<br>*AND*<br>No violation of monotonicity: adequate looking graphs *OR* item scalability >0.30<br>*AND*<br>Adequate model fit:<br>IRT: χ^2 >0.01<br>Rasch: infit and outfit mean squares ≥0.5 and ≤1.5 *OR* Z-standardized values >-2 and <2
	?	Not enough information reported
	-	Criteria for '+' not met

Internal consistency	+	At least low evidence for sufficient unidimensionality<br>*AND*<br>Cronbach's alpha ≥0.70
	?	Criteria for "at least low evidence for sufficient unidimensionality" not met<br>*OR*<br>Evidence for insufficient unidimensionality<br>*OR*<br>Not enough information reported
	-	At least low quality evidence for sufficient unidimensionality<br>*AND*<br>Cronbach's alpha <0.70
Cross-cultural validity\ measurement invariance	+	No important differences found between group factors (such as age, gender, language) in multiple group factor analysis *OR* no important DIF for group factors (McFadden's R^2 <0.02)
	?	Not enough information reported
	-	Important differences between group factors *OR* DIF was found
Reliability	+	ICC or (weighted) kappa or Pearson/Spearman correlation ≥0.70
	?	Not enough information reported
	-	ICC or (weighted) kappa or Pearson/Spearman correlation <0.70
Measurement error	+	SDC or LoA <MIC
	?	MIC not defined *OR* not enough information reported
	-	SDC or LoA > MIC
Criterion validity	+	Correlation with gold standard ≥0.70 *OR* AUC ≥0.70
	?	Not enough information reported
	-	Correlation with gold standard <0.70 *OR* AUC <0.70
Hypotheses testing for construct validity	+	≥75% of the results is in accordance with predefined hypotheses
	?	No relevant results were found
	-	≥75% of the results deviates from predefined hypotheses
Responsivenes s	+	≥75% of the results is in accordance with predefined hypotheses *OR* AUC ≥0.70
	?	No relevant results were found
	-	≥75% of the results deviates from predefined hypotheses *OR* AUC <0.70

AUC = area under the curve, CFA = confirmatory factor analysis, CFI = comparative fit index, CTT = classical test theory, DIF = differential item functioning, EFA = exploratory factor analysis, ICC = intraclass correlation coefficient, IRT = item response theory, LoA = limits of agreement, MIC = minimal important change, PCA = principal component analyses, RMSEA: Root Mean Square Error of Approximation, SEM = Standard Error of Measurement, SDC = smallest detectable change, SRMR: Standardized Root Mean Residuals, TLI = Tucker-Lewis index

Supplementary Table 3. A priori hypotheses for construct validity of the S-QoL instruments.

Hypothesis	Rationale
H1. Correlations between S-QoL scores and other quality-of-life instruments (WHOQOL-BREF, QoLI, EuroQol, SF-36 vitality and mental health domains) will be strong ($r \geq 0.50$).	These instruments assess closely related aspects of health-related quality of life and should therefore show strong positive associations.
H2. Correlations between S-QoL scores and clinician-rated functioning or recovery measures (e.g., GAF, RAS) will be moderate to strong ($r \geq 0.30$).	Better functioning and recovery are expected to be associated with better quality of life.
H3. Correlations between S-QoL scores and psychopathology measures (e.g., PANSS, CGI) will be moderate and negative ($r \leq -0.30$).	Greater symptom severity is expected to be associated with poorer quality of life.
H4. Correlations between S-QoL scores and depressive symptom measures (e.g., CDSS) will be moderate and negative ($r \leq -0.30$).	More severe depressive symptoms are expected to be associated with lower quality of life.
H5. Correlations between S-QoL scores and theoretically less-related constructs (e.g., extrapyramidal symptoms measured by ESRS) will be weak (	$r < 0.30$). These constructs are expected to have a limited relationship with quality of life.
H6. Known groups differing in clinical status (e.g., remission vs. non-remission, high vs. low symptom burden, high vs. low functioning) will differ by at least 0.5 SD or 10% in S-QoL scores.	Clinically distinct groups should demonstrate meaningful differences in perceived quality of life.

Abbreviations: CGI, Clinical Global Impression; CDSS, Calgary Depression Scale for Schizophrenia; ESRS, Extrapyramidal Symptom Rating Scale; GAF, Global Assessment of Functioning; PANSS, Positive and Negative Syndrome Scale; QoLI, Quality of Life Interview; RAS, Recovery Assessment Scale; SF-36, 36-Item Short Form Health Survey; S-QoL, Schizophrenia Quality of Life questionnaire; WHOQOL-BREF, World Health Organization Quality of Life-BREF.

Supplementary Table 4. A priori hypotheses for responsiveness of the S-QoL instruments.

Hypothesis	Rationale
H1. Patients demonstrating clinical improvement according to an external anchor (e.g., CGI improvement, symptom remission) will show a moderate or large improvement in S-QoL scores (effect size ≥ 0.50).	Improvements in clinical status should be reflected by improvements in quality of life.
H2. Patients classified as clinically unchanged will show minimal changes in S-QoL scores (effect size < 0.20).	Stable clinical status should not result in substantial changes in quality-of-life scores.
H3. Improvements in S-QoL scores will correlate moderately to strongly (r ≥ 0.50) with improvements in other quality-of-life measures.	Changes in closely related constructs should occur in the same direction.
H4. Patients achieving symptom remission will demonstrate larger improvements in S-QoL scores than patients who do not achieve remission.	Better symptom control is expected to result in better quality of life.

Abbreviations: CGI, Clinical Global Impression; S-QoL, Schizophrenia Quality of Life questionnaire.

Supplementary Table 5. A list of the excluded studies and reasons for their exclusion.

	Studies	Exclusion Reason
1	Alessandrini M, Lançon C, Fond G, Faget-Agius C, Richieri R, Faugere M, Metairie E, Boucekine M, Llorca PM, Auquier P, Boyer L. A structural equation modelling approach to explore the determinants of quality of life in schizophrenia. Schizophrenia research. 2016 Mar 1;171(1-3):27-34.	No outcome of interest
2	Boyer L, Fernandes S, Faugere M, Richieri R, Auquier P, Fond G, Lancon C. The validity of the SQoL-18 in patients with Bipolar and Depressive Disorders: A psychometric study from the PREMIUM project. Journal of Clinical Medicine. 2022 Jan 29;11(3):743.	No outcome of interest
3	Girard V, Tinland A, Boyer L, Auquier P, French Housing First Study Group. Psychometric properties of the recovery measurement in homeless people with severe mental illness. Schizophrenia research. 2015 Dec 1;169(1-3):292-7.	No outcome of interest
4	Girard V, Tinland A, Boucekine M, Loubière S, Lancon C, Boyer L, Auquier P, French Housing First Study Group. Validity of a common quality of life measurement in homeless individuals with bipolar disorder and schizophrenia. Journal of Affective Disorders. 2016 Nov 1;204:131-7.	No outcome of interest
5	Llorca PM, Gorwood P. Quality of life and schizophrenia: Which evaluation scale for which quality of life?. L'encephale. 2016 Jul 4;42(4):374-8.	Review

Supplementary Table 6. Characteristics of studies on PROM development and content validity.

PROM	Ref #	Phase	Patients						Professionals		
			Sample characteristics			Disease characteristics		Provided input	Sample characteristics		Provided input
			N	Age, mean (SD) [range]	Female (%)	Disease	Severity		N	Professional background	
S-QoL41	Auquier, 2003	Development	60	37 (11)	30	SZ	Mixed severity levels	Face-to-face interviews, non-directive interviews, semi-structured interviews, cognitive interviews, item relevance, comprehensiveness, comprehensibility, face validity	NR	Psychometric analyses	Concept elicitation, Relevance, comprehensiveness, comprehensibility
S-QoL18	Boyer, 2010	Content validity assessment / Item reduction	517	36 (11)	30	SZ	Mixed severity levels	Expert review during item reduction; assessment of relevance across demographic and clinical subgroups; DIF analyses	517	Psychometric analyses	Relevance, comprehensiveness, comprehensibility

	Baumstark, 2013	Content validity in cognitively impaired patients	113	38 (11)	30	SZ	Mixed severity levels	Assessment of item relevance and understandability in patients with cognitive impairment; evaluation of dimensionality and internal structure	NR	NR	Assessment of item relevance and comprehensibility in cognitively impaired patients
	Baumstark, 2014	Content validity in cognitively impaired patients	113	38 (11)	NR	SZ	Mixed severity levels	Assessment of item relevance and understandability in patients with cognitive impairment; evaluation of dimensionality and internal structure	NR	NR	Assessment of item relevance and comprehensibility in cognitively impaired patients
S-QoL-MCAT	Michel, 2018		517	36 (11)	30	SZ	Mixed severity levels	No new content validity assessment; retained original S-QoL41 item bank and wording	NA	NA	No direct content validity study conducted; evidence inherited from S-QoL41 item bank

Abbreviations: DM2: diabetes mellitus type 2; N: number; NR: not reported; NA: not applicable; SD: standard deviation; SZ: Schizophrenia
Blank space: target population not involved

Supplementary Table 7. Results of Measurement Properties (Part A).

PROM	Structural validity[a]			Cross-cultural validity\ measurement invariance			Internal consistency[d]			Reliability Test-retest[e]			Reliability Inter-Rater[e]		
	n	*Q*[b]	*R*[c]	*n*	*Q*[b]	*R*[c]	*n*	*Q*[b]	*R*[c]	*n*	*Q*[b]	*R*[c]	*n*	*Q*[b]	*R*[c]
Auquier, 2003	207	VG	EFA, 8 factors, PsW, SE, Rfa, RFr, RE, PhW, AU, and SL, Varimax rotation, 52% variance explained (+)	-	-	-	207	VG	PsW: 0.92<br>SE: 0.87<br>Rfa: 0.86<br>RFr: 0.84<br>RE: 0.78<br>PhW: 0.75<br>AU: 0.72<br>SL: 0.77<br>Index: 0.94<br>(+)	207	VG	0.79 (+)	-	-	-
Auquier, 2013	236	VG	CFA, 8 factors, PsW, SE, Rfa, RFr, RE, PhW, AU, and SL, RMSEA = 0.035,<br>CFI = 0.95, GFI = 0.99 and SRMR = 0.015 (+)<br>Rasch/INFIT<br>PsW: 0.90-1.09<br>SE: 0.94-1.05	236	VG	DIF tested, items behaved similarly between homeless and non-homeless (+)	236	VG	PsW: 0.73<br>SE: 0.65<br>Rfa: 0.81<br>RFr: 0.80<br>RE: 0.65<br>PhW: 0.84<br>AU: 0.88<br>SL: 0.60	236	VG	0.42–0.77, Rasch model fit (+)	-	-	-

PROM	Structural validity[a]			Cross-cultural validity\ measurement invariance			Internal consistency[d]			Reliability Test-retest[e]			Reliability Inter-Rater[e]		
	n	*Q*[b]	*R*[c]	*n*	*Q*[b]	*R*[c]	*n*	*Q*[b]	*R*[c]	*n*	*Q*[b]	*R*[c]	*n*	*Q*[b]	*R*[c]
			RFa: 0.97-0.99<br>RFr: 0.98-1.00<br>RE: 0.91-1.04<br>PhW: 0.99-1.00<br>AU: 0.98-0.99<br>SL: 0.98-1.00						(+)						
Baumstarck, 2013	113	VG	CFA, 8 factors, PsW, SE, Rfa, RFr, RE, PhW, AU, and SL, (+)<br>Rasch/INFIT<br>Rasch/INFIT<br>**STROOP:**<br>Not impaired:<br>PsW: 0.79-1.18<br>SE: 0.96-0.99<br>Rfa: 0.82-0.99<br>RFr: 0.94-0.96<br>RE: 0.71-1.34<br>PhW: 0.95-0.97	-	-	-	113	VG	**Not impaired:**<br>PsW: 0.70<br>SE: 0.82<br>Rfa: 0.79<br>RFr: 0.87<br>RE: 0.75<br>PhW: 0.79<br>AU: 0.89<br>SL: 0.84<br>**Impaired:**<br>PsW: 0.60<br>SE: 0.79	-	-	-	-	-	-

PROM	Structural validity[a]			Cross-cultural validity\ measurement invariance			Internal consistency[d]			Reliability Test-retest[e]			Reliability Inter-Rater[e]		
	n	*Q*[b]	*R*[c]	*n*	*Q*[b]	*R*[c]	*n*	*Q*[b]	*R*[c]	*n*	*Q*[b]	*R*[c]	*n*	*Q*[b]	*R*[c]
			AU: 0.90-0.91<br>SL: 0.96-1.00<br>**Impaired:**<br>PsW: 0.82-1.07<br>SE: 0.97-0.98<br>Rfa: 0.93-1.00<br>RFr: 0.95-1.00<br>RE: 0.75-1.39<br>PhW: 0.94-0.99<br>AU: 0.91-0.94<br>SL: 1.00-1.05<br>**FLUENCY:**<br>Not impaired<br>PsW: 0.85-1.13<br>SE: 0.96-0.98<br>Rfa: 0.97-0.97<br>RFr: 0.96-0.99<br>RE: 0.94-1.03						Rfa: 0.91<br>RFr: 0.75<br>RE: 0.76<br>PhW: 0.81<br>AU: 0.83<br>SL: 0.64 (+)						

PROM	Structural validity[a]			Cross-cultural validity\ measurement invariance			Internal consistency[d]			Reliability Test-retest[e]			Reliability Inter-Rater[e]		
	n	*Q*[b]	*R*[c]	*n*	*Q*[b]	*R*[c]	*n*	*Q*[b]	*R*[c]	*n*	*Q*[b]	*R*[c]	*n*	*Q*[b]	*R*[c]
			PhW: 0.96-0.99<br>AU: 0.95-1.00<br>SL: 0.96-1.00<br>**Impaired**<br>PsW: 0.93-1.09<br>SE: 0.95-0.98<br>Rfa: 0.92-0.99<br>RFr: 0.88-1.15<br>RE: 0.62-1.27<br>PhW: 0.96-0.98<br>AU: 0.71-0.72<br>SL: 0.01-0.97<br>**TMT**<br>**Not impaired**<br>PsW: 0.91-1.04<br>SE: 0.96-0.99<br>Rfa: 0.96-0.97<br>RFr: 0.97-0.99												

PROM	Structural validity[a]			Cross-cultural validity\ measurement invariance			Internal consistency[d]			Reliability Test-retest[e]			Reliability Inter-Rater[e]		
	n	*Q*[b]	*R*[c]	*n*	*Q*[b]	*R*[c]	*n*	*Q*[b]	*R*[c]	*n*	*Q*[b]	*R*[c]	*n*	*Q*[b]	*R*[c]
			RE: 0.85-1.21<br>PhW: 0.97-0.99<br>AU: 0.95-1.01<br>SL: 1.01-1.04<br>**Impaired**<br>PsW: 0.73-1.15<br>SE: 0.90-1.00<br>Rfa: 0.92-0.93<br>RFr: 0.92-1.00<br>RE: 0.59-1.27<br>PhW: 0.93-1.02<br>AU: 0.74-0.77<br>SL: 0.92-0.97												
Baumstarck, 2014	113	VG	CFA, 8 factors, PsW, SE, Rfa, RFr, RE, PhW, AU, and SL, (+)<br>**Low-impairment**<br>PsW: 0.89-1.17<br>SE: 0.98-1.01	-	-	-	113	VG	**Low-impairment**<br>PsW: 0.76<br>SE: 0.85<br>Rfa: 0.88<br>RFr: 0.8	-	-	-	-	-	-

PROM	Structural validity[a]			Cross-cultural validity\ measurement invariance			Internal consistency[d]			Reliability Test-retest[e]			Reliability Inter-Rater[e]		
	n	*Q*[b]	*R*[c]	*n*	*Q*[b]	*R*[c]	*n*	*Q*[b]	*R*[c]	*n*	*Q*[b]	*R*[c]	*n*	*Q*[b]	*R*[c]
			Rfa: 0.89-0.9<br>RFr: 0.92-1.02<br>RE: 0.74-1.19<br>PhW: 0.95-0.99<br>AU: 0.94-0.95<br>SL: 0.93-0.94<br><br>**High impairment**<br>PsW: 0.98-1.08<br>SE: 0.97-0.99<br>Rfa: 0.97-0.98<br>RFr: 0.97-1<br>RE: 0.81-1.2<br>PhW: 0.95-0.98<br>AU: 0.95-0.99<br>SL: 0.98-1.04						RE: 0.82<br>PhW: 0.61<br>AU: 0.84<br>SL: 0.88<br><br>**High impairment**<br>PsW: 0.63<br>SE: 0.81<br>Rfa: 0.87<br>RFr: 0.81<br>RE: 0.73<br>PhW: 0.86<br>AU: 0.88<br>SL: 0.65 (+)						

PROM	Structural validity[a]			Cross-cultural validity\ measurement invariance			Internal consistency[d]			Reliability Test-retest[e]			Reliability Inter-Rater[e]		
	n	*Q*[b]	*R*[c]	*n*	*Q*[b]	*R*[c]	*n*	*Q*[b]	*R*[c]	*n*	*Q*[b]	*R*[c]	*n*	*Q*[b]	*R*[c]
Boyer, 2010	517	VG	PCA, 8 factors, PsW, SE, Rfa, RFr, RE, PhW, AU, and SL, (+)<br>PsW: 0.91-1.05<br>SE: 0.98-0.99<br>RFa: 0.96-1.02<br>RFr:0.98-1.00<br>RE: 0.96-1.03<br>PhW: 0.97-0.99<br>AU: 0.98-0.99<br>SL: 0.98-1.01	517	VG	DIF tested, items behaved similarly between gender, age, education, clinical form (+)	517	VG	PsW: 0.73<br>SE: 0.74<br>RFa: 0.81<br>RFr: 0.73<br>RE: 0.74<br>PhW: 0.79<br>AU: 0.84<br>SL: 0.72<br>Index: 0.88 (+)	517	VG	PsW: 0.70<br>SE: 0.78<br>RFa: 0.70<br>RFr:0.75<br>RE: 0.73<br>PhW: 0.76<br>AU: 0.63<br>SL: 0.50<br>Index: 0.73 (+)	-	-	-
Caqueo-Urizar, 2014	253	VG	EFA, 8 factors, PsW, SE, Rfa, RFr, RE, PhW, AU, and SL (+)<br>Rasch/INFIT min-max Bolivia<br>PsW: 0.85-1.19<br>SE: 0.84-1.01<br>RFa: 0.88-0.88<br>RFr:0.95-0.99	253	VG	Cross-cultural validity was rated as sufficient. Differential item functioning analysis across countries (Peru, Bolivia, and	550	VG	**Bolivia**<br>PsW: 0.81<br>SE: 0.49<br>RFa: 0.98<br>RFr:0.62<br>RE: 0.38<br>PhW: 0.90<br>AU: 0.87	93	VG	0.74-0.86 (+)	-	-	-

PROM	Structural validity[a]			Cross-cultural validity\ measurement invariance			Internal consistency[d]			Reliability Test-retest[e]			Reliability Inter-Rater[e]		
	n	*Q*[b]	*R*[c]	*n*	*Q*[b]	*R*[c]	*n*	*Q*[b]	*R*[c]	*n*	*Q*[b]	*R*[c]	*n*	*Q*[b]	*R*[c]
			RE: 0.90-1.59<br>PhW: 0.97-1.00<br>AU: 0.98-0.99<br>SL: 1.00-1.00<br>Rasch/INFIT min-max Chile<br>PsW: 0.85-1.14<br>SE: 0.89-0.89<br>RFa: 0.82-0.82<br>RFr:0.88-1.04<br>RE: 0.81-1.17<br>PhW: 0.99-1.01<br>AU: 1.00-1.01<br>SL: 0.95-1.04<br>Rasch/INFIT min-max Peru<br>PsW: 0.84-1.07<br>SE: 0.96-1.01<br>RFa: 0.94-0.95			Chile) showed evidence of invariance for 17 of the 18 items, with only one item in the resilience dimension displaying meaningful DIF (+)			SL: 0.44<br>**Chile**<br>PsW: 0.80<br>SE: 0.69<br>RFa: 0.97<br>RFr: 0.91<br>RE: 0.16<br>PhW: 0.62<br>AU: 0.78<br>SL: 0.49<br>**Peru**<br>PsW: 0.75<br>SE: 0.60<br>RFa: 0.87<br>RFr:0.70<br>RE: 0.37<br>PhW: 0.85<br>AU: 0.66						

PROM	Structural validity[a]			Cross-cultural validity\ measurement invariance			Internal consistency[d]			Reliability Test-retest[e]			Reliability Inter-Rater[e]		
	n	*Q*[b]	*R*[c]	*n*	*Q*[b]	*R*[c]	*n*	*Q*[b]	*R*[c]	*n*	*Q*[b]	*R*[c]	*n*	*Q*[b]	*R*[c]
			RFr:0.97-1.00<br>RE: 0.76-1.20<br>PhW: 0.98-0.99<br>AU: 0.95-0.96<br>SL: 0.88-0.91						SL: 0.73 (+)						
Chou, 2011	41	VG	EFA, 8 factors, PsW, SE, Rfa, RFr, RE, PhW, AU, and SL (+)	-	-	-	41	VG	PsW: 0.81<br>SE: 0.84<br>RFa: 0.72<br>RFr: 0.88<br>RE: 0.89<br>PhW: 0.71<br>AU: 0.71<br>SL: 0.44<br>Index: 0.93 (+)	41	VG	0.64–0.87 (+)	-	-	-
Chou, 2015	41	VG	CFA, 8 factors, PsW, SE, Rfa, RFr, RE, PhW, AU, and SL, (+)	-	-	-	477	VG	PsW: 0.60<br>SE: 0.64<br>RFa: 0.31<br>RFr: 0.74	477	VG	PsW: 0.67<br>SE: 0.84<br>RFa: 0.76<br>RFr: 0.58	-	-	-

PROM	Structural validity[a]			Cross-cultural validity\ measurement invariance			Internal consistency[d]			Reliability Test-retest[e]			Reliability Inter-Rater[e]		
	n	*Q*[b]	*R*[c]	*n*	*Q*[b]	*R*[c]	*n*	*Q*[b]	*R*[c]	*n*	*Q*[b]	*R*[c]	*n*	*Q*[b]	*R*[c]
									RE: 0.83<br>PhW: 0.71<br>AU: 0.79<br>SL: 0.44<br>Index: 0.85 (+)			RE: 0.59<br>PhW: 0.78<br>AU: 0.71<br>SL: 0.67<br>Index: 0.87 (+)			
Girard, 2017	55	VG	CFA, 8 factors, PsW, SE, Rfa, RFr, RE, PhW, AU, and SL, (+)<br>PsW: 0.75-1.32<br>SE: 0.73-1.24<br>RFa: 0.65-1.43<br>RFr: 0.75-1.11<br>RE: 0.93-1.17<br>PhW: 0.72-1.36<br>AU: 0.62-1.49<br>SL: 0.88-1.10	-	-	-	55	VG	PsW: 0.65<br>SE: 0.67<br>RFa: 0.86<br>RFr: 0.77<br>RE: 0.72<br>PhW: 0.83<br>AU: 0.86<br>SL: 0.72 (+)	55	VG	0.14–0.87 (+)	-	-	-
Kachouchi, 2026	43	VG	EFA, 3 factors (+)	-	-	-	43	VG	0.866 (+)	-	-	-	-	-	-

PROM	Structural validity[a]			Cross-cultural validity\ measurement invariance			Internal consistency[d]			Reliability Test-retest[e]			Reliability Inter-Rater[e]		
	n	*Q*[b]	*R*[c]	*n*	*Q*[b]	*R*[c]	*n*	*Q*[b]	*R*[c]	*n*	*Q*[b]	*R*[c]	*n*	*Q*[b]	*R*[c]
Michel, 2017	517	VG	CFA, 8 factors, PsW, SE, Rfa, RFr, RE, PhW, AU, and SL, (+)	517	VG	DIF tested, items behaved similarly between gender, insight, paranoia (+)	517	VG	0.88 (+)	517	VG	0.80–0.92 (+)	-	-	-
Reine, 2005	207	VG	CFA, 8 factors, PsW, SE, Rfa, RFr, RE, PhW, AU, and SL, (+)	-	-	-	-	-	-	-	-	-	-	-	-

PsW, Psychological well-being; SE, Self-esteem; Rfa, Family relationship; RFr, Relationships with friends; RE, Resilience; PhW, Physical well-being; AU, Autonomy; SL, Sentimental life.

Supplementary Table 7. Results of Measurement Properties (Part B).

PROM	Measurement error			Criterion validity			Hypotheses testing for construct validity			Responsiveness		
	n	*Q*[b]	*R*[c]	*n*	*Q*[b]	*R*[c]	*n*	*Q*[b]	*R*[c]	*n*	*Q*[b]	*R*[c]
Auquier, 2003	-	-	-	-	-		207	VG	SF-36 (0.39–0.61), QoLI (0.11–0.59), EuroQol (0.48), PANSS (-0.23)<br>SQoL with CDSS (-0.39)<br>SQoL with GAF (0.27)<br>SQoL with CGI (0.32) (+)	207	VG	Responsiveness was rated as indeterminate. Although significant changes and small-to-moderate effect sizes were observed for several S-QoL dimensions in improved patients, no predefined hypotheses regarding expected change were reported. (+)
Auquier, 2013	-	-	-	-	-	-	236	VG	SF-36 (0.21–0.53), MCSI (-0.61 – -20), RAS (0.31–0.64) (+)	115	VG	Responsiveness was rated as sufficient. Sensitivity to change was assessed over 6 months using the MCSI as an external anchor. Significant improvements and small-to-moderate effect sizes were observed in several S-QoL 18 dimensions and the index score among improved patients, while no meaningful changes were found in non-improved patients. (+)
Baumstarck, 2013	-	-	-	-	-	-	113	VG	PANNS (-0.37.5)<br>CDSS (-0.37.5-0.87.5) (+)	-	-	-
Baumstarck, 2014	-	-	-	-	-	-	113	VG	PANNS (-37) (+)	-	-	-
Boyer, 2010	-	-	-	-	-	-	517	VG	SF-36 (13–58), EuroQol (0.50), PANSS (−28) (+)	28	VG	The responsiveness of the S-QoL 18 was assessed in 28 patients whose health improved over 6 months. Significant changes were observed for the Resilience dimension

PROM	Measurement error			Criterion validity			Hypotheses testing for construct validity			Responsiveness		
	n	*Q*[b]	*R*[c]	*n*	*Q*[b]	*R*[c]	*n*	*Q*[b]	*R*[c]	*n*	*Q*[b]	*R*[c]
												and the overall index, with effect sizes greater than 0.2 for Family relationships, Resilience, Physical well-being, Autonomy, and the Index, indicating that the S-QoL 18 is sensitive to clinical changes over time. (+)
Caqueo-Urizar, 2014	-	-	-	-	-	-	253	VG	PANNS (-0.36- -0.38) (+)	-	-	-
Chou, 2011	-	-	-	-	-	-	41	VG	SQLS-R4 (-0.62- -0.74), WHOQoL-BREF (range -0.34– -0.74), RSES (-0.43) (+)	-	-	-
Chou, 2015	-	-	-	-	-	-	41	VG	SQLS-R4 (-0.58- -0.74), WHOQoL-BREF (range -0.35– -0.69), RSES (-0.68) (+)	30	VG	Although direct responsiveness testing was not performed, the S-QoL-18 demonstrated good sensitivity to clinical differences between groups. Patients with symptom remission (N=30) had significantly higher scores on most subscales and the index compared with non-symptom-remission patients (N=30), and their scores were similar to healthy controls (N=30). This indicates that the S-QoL-18 can distinguish between different clinical states, reflecting its potential responsiveness to meaningful changes in health-related quality of life.

PROM	Measurement error			Criterion validity			Hypotheses testing for construct validity			Responsiveness		
	n	*Q*[b]	*R*[c]	*n*	*Q*[b]	*R*[c]	*n*	*Q*[b]	*R*[c]	*n*	*Q*[b]	*R*[c]
Girard, 2017	-	-		-	-	-				-	-	-
Kachouchi, 2026	-	-	-	-	-	-	43	VG	PANSS (-0.341), WHOQOL-BREF(0.649), GAF (0.788)	-	-	-
Michel, 2017	-	-	-	-	-	-	517	VG	PANSS (r= –0.139 - –0.380) (+)	-	-	The SQoL-MCAT demonstrated good responsiveness in detecting changes in patients' quality of life over time. Analyses showed that MCAT-adaptive scores accurately reflected clinical variations, with high correlations (>0.90) between adaptive and full-item scores, and RMSE values below 0.3 for most dimensions, indicating the instrument's sensitivity to changes in health status among patients with schizophrenia. (+)
Reine, 2005	-	-	-	-	-	-	207	VG	PANSS (r=–0.23), CDSS (r=–0.39), CGI (r=–0.29), GAF (r=–0.27), ESRS (r=–0.23) (+)	46	VG	Responsiveness of the S-QoL was demonstrated in 46 patients improving between D0 and D30 according to the CGI. Significant improvements were observed in the S-QoL Index, with high effect sizes for Sentimental Life and moderate ones for Psychological Well-Being and Self-Esteem. Changes in QoLI Overall Life Satisfaction and SF-36 composite scores were not significant, although some subscales showed moderate

PROM	Measurement error			Criterion validity			Hypotheses testing for construct validity			Responsiveness		
	n	*Q*[b]	*R*[c]	*n*	*Q*[b]	*R*[c]	*n*	*Q*[b]	*R*[c]	*n*	*Q*[b]	*R*[c]
												improvements, indicating that S-QoL is sensitive to short-term clinical changes. (+)

[a]Studies with a Risk of Bias (RoB) rating of Inadequate (I) or Doubtful (D) were excluded from analysis and therefore from the final summary results.

[b]Meth qual=Methodological Quality based in COSMIN Risk of Bias Assessment. VG=Very Good; A=Adequate; D=Doubtful; I=Inadequate

[c]Rating of '+'=Sufficient (the result fulfills the COSMIN updated criteria for good measurement properties outlined in Table 1); '?'=Indeterminate (we cannot determine if the result fulfills the criteria as not enough information reported); '-'=Insufficient (the result does not meet the criteria for good measurement properties).

[d]Cronbach's alpha (α) is reported for Internal Consistency results, unless otherwise stated.

[e] Intraclass Correlation Coefficient (ICC) is reported for Internal Consistency results, unless otherwise stated. Studies with a Risk of Bias (RoB) rating of Inadequate (I) or Doubtful (D) were excluded from analysis and therefore from the final summary results.

[f]Pooled results are detailed in the Summary of Findings Table (Appendix I)

SF-36, Short Form 36 Health Survey; QoLI, Quality of Life Interview; EuroQol, EuroQol EQ-5D; PANSS, Positive and Negative Syndrome Scale; CDSS, Calgary Depression Scale for Schizophrenia; GAF, Global Assessment of Functioning; CGI, Clinical Global Impression; MCSI, Mental Component Summary Index; RAS, Recovery Assessment Scale; SQLS-R4, Schizophrenia Quality of Life Scale Revision 4; WHOQoL-BREF, World Health Organization Quality of Life-BREF; RSES, Rosenberg Self-Esteem Scale; BPRS, Brief Psychiatric Rating Scale; ESRS, Extrapyramidal Symptom Rating Scale.

Supplementary Table 8. Hypotheses testing for construct validity: comparisons and results.

Reference	Results	Comparison with other outcome measurement instruments (convergent validity)					Comparison between subgroups (discriminative or know groups validity)				Total number of results in accordance with hypotheses
	**PROM**	**Comparator instrument**	**Construct measured**	**Expected correlation**	**Observed correlation**	**Result in accordance with hypothesis (+ / -)**	**Subgroups compared**	**Expected mean (SD) difference in PROM scores**	**Observed mean (SD) difference in PROM scores**	**Result in accordance with hypothesis (+ / -)**	
Auquier et al., 2003	S-QoL41	SF-36	Health-related quality of life	≥ 0.50	0.39-0.61	1+					4+,3-
		QoLI	Quality of life	≥ 0.50	0.11-0.59	1+					
		EuroQol	Health-related quality of life	≥ 0.50	0.48	1-					
		PANSS	Symptom severity	≤ -0.30	-0.23	1-					
		CDSS	Depressive symptoms	≤ -0.30	-0.39	1+					
		GAF	Global functioning	≥ 0.30	0.27	1-					
		CGI	Clinical severity	≤ -0.30	-0.32	1+					
							Outpatients vs Inpatients	Outpatients > Inpatients (higher QoL expected)	PsW: 64.1 (28.0) vs 54.7 (31.6) RFr: 50.5 (30.4) vs 43.4 (31.4) PhW: 53.0 (26.0) vs 47.9 (27.0) AU: 64.6 (26.3) vs 51.8 (31.6) Index: 57.4 (18.5) vs 53.4 (19.4)	1+	3+

							Higher education (University vs Primary/ High school)	University > lower education	RFa: 70.4 (26.9) vs 60.4 (31.6) RFr: 55.9 (27.3) vs 46.4 (31.5)	1+	
							Gender (Male vs Female)	No difference expected (overall hypothesis: no sociodemographic effect)	RFa: Male 65.7 (28.2) vs Female 58.4 (32.6) SL: Male 38.7 (30.3) vs Female 49.4 (31.2) Other dimensions: non-significant differences	1+	
Number of results in accordance with hypotheses											7+,3-
Chou et al., 2011	S-QoL41	SQLS-R4	Schizophrenia-specific quality of life	≥ 0.50	0.62-0.74	1+					3+
		WHOQOL-BREF	Quality of life	≥ 0.50	0.34-0.74	1+					
		RSES	Self-esteem	≥ 0.30	0.43	1+					
Number of results in accordance with hypotheses											3+
Reine et al. 2005	S-QoL41	PANSS	Symptom severity	≤ -0.30	-0.23	1-					3+,2-
		CDSS	Depressive symptoms	≤ -0.30	-0.39	1+					
		CGI	Clinical severity	≤ -0.30	-0.29	1-					
		GAF	Global functioning	≥ 0.30	-0.27	1+					

		ESRS	Extrapyramidal symptoms	≥ 0.30	-0.23	1+					
							Duration of illness vs QoL	QoL should not depend strongly on illness duration	Weak correlations only (e.g., Resilience r = −0.20)	1+	5+
							Duration of neuroleptic treatment vs QoL	QoL should not depend strongly on treatment duration	Weak negative correlation with Resilience (r = −0.18)	1+	
							First-episode vs multi-episode schizophrenia	No major differences expected	No significant differences observed	1+	
							Schizophrenia subtype vs QoL	No major differences expected	No significant differences observed	1+	
							Relapse vs stabilized patients	Relapsing patients should report poorer QoL	Lower SF-36 Social Functioning, Mental Health, and Mental Composite scores in relapsing patients	1+	
Number of results in accordance with hypotheses											8+,2-
	S-QoL18	SF-36	Health-related quality of life	≥ 0.50	0.21-0.53	1-					1+, 2-

Auquier et al., 2013		MSCI	Clinical status	0.3-0.49	-0.61--0.20	1-					
		RAS	Recovery	≤ 0.30	0.31-0.64	1+					
							Homeless vs Non-homeless patients with schizophrenia	Homeless patients expected to report lower QoL than non-homeless patients	All dimensions lower in homeless group: PsW, SE, RFa, RFr, RE, PhW, AU, SL, Index (exact means not fully reported in text; significant differences for SE, RFa, RFr, RE, SL and Index)	1+	4+
							Clinically improved vs non-improved patients (MCSI-based)	Improved patients expected to show higher QoL over time	Significant improvement at 6 months in PsW, SE, RFa, SL, Index; ES > 0.2 in PsW, SE, RFa, RFr, RE, SL, Index	1+	
							Age and sociodemographics	No major association expected	No significant associations except minimal effects	1+	
							Clinical severity (MCSI)	Higher severity → lower QoL	Correlations range: r = −0.61 to −0.20 across dimensions (strongest PsW and SE)	1+	
Number of results in accordance with hypotheses											5+, 2-

Baumstarck et al. 2013	S-QoL18	PANSS	Symptom severity	≤ -0.30	-0.37.5	1+					2+
		CDSS	Depressive symptoms	≤ -0.30	-0.37--0.87	1+					
							Age (younger vs older)	No significant difference expected	No significant difference (r < 0.30, NS)	1+	4+
							Gender (male vs female)	No significant difference expected	Mostly no significant differences (except RFa: males higher)	1+	
							Educational level (low vs high)	No significant difference expected	No consistent significant differences	1+	
							Duration of illness (short vs long)	No significant difference expected	No significant correlation (r < 0.30, NS)	1+	
Number of results in accordance with hypotheses											6+
Baumstarck et al. 2014	S-QoL18	PANSS	Symptom severity	≤ -0.30	-0.37	1+					1+
							age (younger vs older / no–low vs high impairment)	No significant effect expected	Age almost never correlated with QoL dimensions	1+	5+

							Gender (men vs women)	Small or no differences expected	Women generally lower QoL scores except sentimental/sexual life	1+	
							Educational level (<12 vs ≥12 years)	No major differences expected	Moderately satisfactory: small effect sizes (<0.2 in many dimensions)	1+	
							Disease duration (short vs long)	No correlation expected	Almost no correlations with QoL dimensions	1+	
							Cognitive status (no/low vs highly impaired)	Lower QoL expected in highly impaired group	Lower construct validity + differences in factor stability; suitability index 78–82%	1+	
Number of results in accordance with hypotheses											6+
Boyer et al. 2010	S-QoL18	SF-36	Health-related quality of life	≥ 0.50	0.13-0.58	1-					1+, 2-
		EuroQol	Health-related quality of life	≥ 0.50	0.50	1+					
		PANSS	Symptom severity	≤ -0.30	-0.28	1-					
							Inpatients vs Outpatients	Outpatients > Inpatients	PsW: 64.1 (28.0) vs 54.7 (31.6);	1+	3+, 1-

								(higher QoL expected)	RFr: 50.5 vs 43.4; PhW: 53.0 vs 47.9; AU: 64.6 vs 51.8; Index: 57.4 vs 53.4		
							Higher education vs lower education	Higher education > lower QoL (expected higher QoL)	RFa: 70.4 (26.9) vs 60.4 (31.6); RFr: 55.9 (27.3) vs 46.4 (31.5)	1+	
							Male vs Female	No significant difference expected	RFa: 65.7 vs 58.4 (p=0.01); SL: 38.7 vs 49.4 (p<0.01); others non-significant	1−	
							Stable illness duration groups	No difference expected	No significant differences except weak correlation RE (r = −0.21)	1+	
Number of results in accordance with hypotheses											4+,3-
Caqueo-Urizar et al. 2014	S-QoL18	PANSS	Symptom severity	≤ -0.30	-0.36--0.38	1+					1+
							Country differences (Bolivia vs Chile vs Peru)	Some variability expected but overall similar structure	8-factor structure generally replicated; minor	1+	5+

									differences (SE not retrieved in Bolivia)		
							Gender (men vs women)	No or small differences expected	Mostly non-significant; few exceptions (RFa higher in men in Bolivia & Chile)	1+	
							Educational level (low vs high)	Higher education → slightly higher QoL expected	Mixed but some significant differences (e.g., PsW, RFr, Index in Peru/Bolivia)	1+	
							Age	No correlation expected	Almost all correlations < 0.3 or non-significant (except one case: RFa in Chile)	1+	
							Disease duration	No correlation expected	Almost all correlations < 0.3 or non-significant	1+	
Number of results in accordance with hypotheses											6+
Chou et al. 2015	S-QoL18	SQLS-R4	Schizophrenia-specific quality of life	≥ 0.50	0.58-0.74	1+					3+
		WHOQOL-BREF	Quality of life	≥ 0.50	0.35-0.69	1+					
		RSES	Self-esteem	≥ 0.30	0.68	1+					
							Symptom-remission vs Non-symptom-remission	Patients in symptom remission should report higher QoL	Significant differences found for S-QoL-18-C Index and 7 of 8 dimensions	1+	4+

							schizophrenia patients	scores than non-remission patients	(all except Psychological Well-being); remission group had substantially higher QoL scores		
							Symptom-remission patients vs Healthy individuals	QoL scores expected to be similar between remission patients and healthy controls	No significant differences between remission group and healthy individuals on Index and 7 dimensions	1+	
							Non-symptom-remission patients vs Healthy individuals	Non-remission patients expected to have lower QoL than healthy controls	Non-remission group had significantly lower scores than healthy controls on Index and 7 dimensions	1+	
							Patients with ≥3 hospitalizations vs <3 hospitalizations	More hospitalizations expected to be associated with poorer QoL	Significant differences for Relationships with Friends (p=0.016), Sentimental Life (p=0.030), and SQLS-R4 Psychosocial, Vitality and Total scores	1+	
Number of results in accordance with hypotheses											7+
Kachouchi et al. 2026	S-QoL18	GAF	Global functioning	≥ 0.30	0.78	1+					3+

		PANSS	Symptom severity	≤ -0.30	-0.341	1+					
		WHOQOL-BREF	Quality of life	≥ 0.50	0.649	1+					
							Age vs QoL	QoL should not be strongly related to age	Non-significant correlation (r = 0.150, p = 0.336)	1+	2+
							Duration of illness vs QoL	QoL should not depend strongly on illness duration	Non-significant correlation (r = -0.089, p = 0.572)	1+	
Number of results in accordance with hypotheses											5+
Michel et al. 2017	S-QoL-MCAT	PANSS	Symptom severity	≤ -0.30	-0.13--0.38	1-					1-
							Duration of illness vs QoL	QoL should not be strongly associated	SQoL-MCAT dimension scores were not correlated with	1+	3+

								with illness duration	duration of illness		
							Gender invariance	QoL items should function similarly across males and females	Only 3 items showed negligible DIF; no moderate or large DIF detected	1+	
							Paranoid symptoms invariance	Item functioning should not differ according to paranoid symptom status	Only 1 item showed negligible DIF; overall invariance maintained	1+	
Number of results in accordance with hypotheses											3+,1-

Abbreviations: CGI, Clinical Global Impression; CDSS, Calgary Depression Scale for Schizophrenia; ESRS, Extrapyramidal Symptom Rating Scale; GAF, Global Assessment of Functioning; PANSS, Positive and Negative Syndrome Scale; QoLI, Quality of Life Interview; RAS, Recovery Assessment Scale; SF-36, 36-Item Short Form Health Survey; S-QoL, Schizophrenia Quality of Life questionnaire; WHOQOL-BREF, World Health Organization Quality of Life-BREF.

Supplementary Table 9. Hypotheses testing for responsiveness: comparisons and results.

**Reference**	**Results**			**Construct approach**									**Total number of results in accordance with hypothesis**
				**Comparison with other outcome measurement instruments**					**Before and after intervention**				
	PROM	Intervention	Time interval	Comparator instrument	Construct measured	Expected correlation	Observed correlation	Result in accordance with hypothesis (+ / -)	Intervention	Expected difference	Observed difference	Result in accordance with hypothesis (+ / -)	
Auquier, 2003	S-QoL41	Clinical improvement (46 improved patients according to CGI)	Baseline to Day 30						Responsiveness of S-QoL Index	ES≥ 0.20	S-QoL Index ES = 0.44; significant improvement ($p < 0.05$)	1+	7+, 2-
									Psychological well-being (PsW)	ES≥ 0.20	ES = 0.35; significant improvement ($p < 0.05$)	1+	
									Self-esteem (SE)	ES≥ 0.20	ES = 0.28; significant improvement ($p < 0.05$)	1+	
									Sentimental life (SL)	ES≥ 0.20	ES = 0.41; significant improvement ($p < 0.01$)	1+	
									Family relationships (RFa)	ES≥ 0.20	ES = 0.18; non-significant	1-	

									Relationships with friends (RFr)	ES≥ 0.20	ES = 0.29; non-significant	1+	
									Resilience (RE)	ES≥ 0.20	ES = 0.12; non-significant	1-	
									Physical well-being (PhW)	ES≥ 0.20	ES = 0.32; non-significant	1+	
									Autonomy (AU)	ES≥ 0.20	ES = 0.10; non-significant	1-	
Number of results in accordance with hypotheses													7+, 2-
Reine, 2005	S-QoL41	Clinical improvement (46 improved patients according to CGI)	Baseline to Day 30	CGI improvement anchor	Change in QoL	r ≥ 0.50	PANSS total: ES = −0.41; CDSS: ES = −0.45	1+					
									S-QoL Index	ES≥ 0.20	Not numerically reported (significant improvement stated only)		
									Psychological Well-being	ES≥ 0.20	Moderate effect size (value not reported)		
									Self-Esteem	ES≥ 0.20	Moderate effect size		

											(value not reported)		
									Sentimental Life	ES≥ 0.20	High effect size (value not reported)		
									Social Relations (RFa/RFr combined mention)	ES≥ 0.20	High effect size (value not reported)		
									Resilience	ES≥ 0.20	Moderate effect size (value not reported)		
Number of results in accordance with hypotheses													1+
Boyer, 2010	S-QoL18	Clinical improvement (patients improved at 6 months, n=28)	Baseline to 6 months						Responsiveness (QoL change)	ES≥ 0.20	Index > 0.2; RFa > 0.2; RE > 0.2; PhW > 0.2; AU > 0.2	1+	3+
									Resilience (RE)	ES≥ 0.20	Significant difference (p < 0.05); ES not numerically reported (only stated >0.2)	1+	
									Global Index	ES≥ 0.20	Significant difference (p < 0.05); ES > 0.2	1+	
Number of results in accordance with hypotheses													3+
Auquier, 2013	S-QoL18	Clinical improvement (patients improved at 6 months, n=90)	Baseline to 6 months						Sensitivity to change	ES≥ 0.20	Improved group: ES > 0.2 for PsW, SE, RFa, RFr, RE, SL, Index	1+	4+

									Psychological Well-being (PsW)	ES≥ 0.20	ES > 0.2 (significant p<0.05)	1+	
									Self-esteem (SE)	ES≥ 0.20	ES > 0.2 (significant p<0.05)	1+	
									Social / emotional domains (SL, RFa, RFr, RE)	ES≥ 0.20	ES > 0.2 (only SL also in non-improved group)	1+	
Number of results in accordance with hypotheses													4+
Chou, 2015	S-QoL18	Clinical status groups (symptom remission vs non-remission vs healthy)	NR						Group comparison (ANOVA)	ES≥ 0.20	Index + 7 subscales: significant ANOVA (p = 0.001); non-remission < remission ≈ healthy; remission ≈ healthy (no difference)	1+	1+
Number of results in accordance with hypotheses													1+

ES: Effect Size; NR: Not Reported; SRM: Standardized Response Mean.

Supplementary Table 10. Summary of findings with the evidence for each measurement property for each PROM*.

Measurement property	S-QoL41		S-QoL18		S-QoL-MCAT	
	Overall rating	Certainty of evidence	Overall rating	Certainty of evidence	Overall rating	Certainty of evidence
Content validity						
Relevance	+	High	+	Moderate	+	Very low
Comprehensiveness	+	High	+	Moderate	+	Very low
Comprehensibility	+	High	+	High	+	Very low
Structural validity	+	High	+	High	+	High
Internal consistency	+	High	+	High	+	High
Cross-cultural validity	?	Very low	+	Low	+	Very low
Measurement invariance	+	Very low	+	Moderate	+	Moderate
Reliability	+	High	+	High	+	Very low
Measurement error	?	Very low	?	Very low	?	Very low
Criterion validity		NR		NR		NR
Construct: known groups	+	High	+	High	+	Moderate
Construct: other instruments	+	High	+	High	+	Moderate
Responsiveness	+	High	+	High	+	Very low

* Colors represent sufficiency of measurement properties, shading represents quality of the evidence:

Green: sufficient; red: insufficient yellow: inconsistent; grey: indeterminate; darker shading: higher quality evidence

Blank space: lack of evidence
